# Engineering Biomimetic Microvascular Capillary Networks in Hydrogel Fibrous Scaffolds via Microfluidics-Assisted Co-Axial Wet-Spinning

**DOI:** 10.1021/acsami.4c15221

**Published:** 2024-11-20

**Authors:** Alessia Paradiso, Marina Volpi, Diana C. Martinez, Jakub Jaroszewicz, Marco Costantini, Wojciech Swieszkowski

**Affiliations:** † Faculty of Materials Sciences and Engineering, Warsaw University of Technology, Warsaw 02-507, Poland; ‡ Institute of Physical Chemistry, Polish Academy of Sciences, Warsaw 01-224, Poland

**Keywords:** biofabrication, 3D bioprinting, microfluidic-assisted wet-spinning, hydrogel fibers, microvascular tissue engineering, blood capillary network

## Abstract

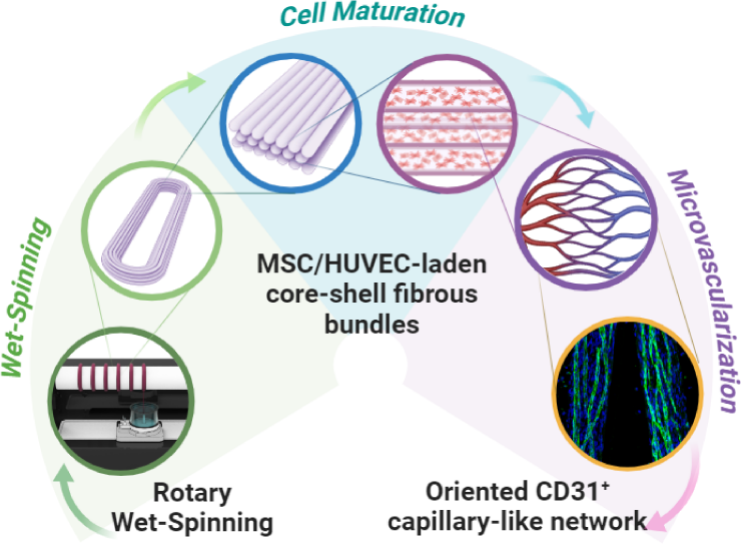

The microvascular bed plays a crucial role in establishing nutrient exchange and waste removal, as well as maintaining tissue metabolic activity in the human body. However, achieving microvascularization of engineered 3D tissue constructs is still an unsolved challenge. In this work, we developed biomimetic cell-laden hydrogel microfibers recapitulating oriented microvascular capillary-like networks by using a 3D bioprinting technique combined with microfluidics-assisted coaxial wet-spinning. Highly packed and aligned bundles embedding a coculture of human bone marrow-derived mesenchymal stem cells (MSCs) and human umbilical vein endothelial cells (HUVECs) were produced by simultaneously extruding two different bioinks. To this aim, core–shell fibers were wet-spun in a coagulation bath to collect the scaffolds later on a rotary drum. Initially, the versatility of the proposed system was assessed for the extrusion of multimaterial core–shell hydrogel fibers. Subsequently, the platform was validated for the *in vitro* biofabrication of samples promoting optimal cell alignment along the fiber axis. After 3 weeks of culture, such fiber configuration resulted in the development of an oriented capillary-like network within the fibrin-based core and in the endothelial-specific CD31 marker expression upon MSC/HUVEC maturation. Synergistically, the vertical arrangement of the coaxial nozzle coupled with the rotation of the fiber collector facilitated the rapid creation of tightly packed bundles characterized by a dense, oriented, and extensively branched capillary network. Notably, such findings suggest that the proposed biofabrication strategy can be used for the microvascularization of tissue-specific 3D constructs.

## 1 Introduction

Blood vessels serve as vital conduits for maintaining tissue homeostasis by delivering oxygen and nutrients, along with removing metabolic waste products.

On the one hand, there are large-diameter vessels (>8 mm, large arteries) and medium-size conduits (6–8 mm, e.g., femoral artery and carotid), which promote the efficient transport of fluids throughout the body. On the other hand, small-diameter vessels (<6 mm) consist of blood vessels typically less than 300 μm in diameter, which form the microvascular network, also known as the microvascular bed. At the functional level, arterioles and venules (1–100 μm) regulate the blood flow through the body, while capillaries (4–10 μm) facilitate the rapid exchange of fluid, solutes, and cells across the endothelial layer at the tissue level.^1−3^

Given that diffusion alone inherently limits nutrient and oxygen penetration (<100–200 μm), the microvascular network performs efficiently, thanks to its high density and distribution all over the human body, by delivering molecules to and from cells.

For this reason, the ability to effectively integrate a microvasculature network within bioartificial 3D constructs is of paramount importance to mimic the native tissue environment and ensure their functionality. However, such strict requirements impose constraints in developing thicker and physiologically relevant engineered tissue structures. Recent advances in the biofabrication field have successfully recapitulated blood vessels ranging from arteries and veins^4−6^ to arterioles and venules.^7,8^ In this framework, 3D (bio)printing (3DBP) has allowed researchers to produce spatially controlled high-resolution scaffolds by layer-by-layer deposition of hydrogel-based biomaterials.^9,10^ Such an approach enabled the precise encapsulation of cells within the 3D constructs, providing them extracellular matrix (ECM) cues and structural support in a controlled and precise way. A broad range of 3DBP strategies has been used to recreate *in vitro* prevascularized scaffolds. Among them, multimaterial extrusion^8,11^ and light-based techniques, such as stereolithography^12,13^ and volumetric 3DBP,^14,15^ have been widely tested. Favorably, 3DBP offers high repeatability and scale-up possibilities for the fabrication of prevascularized constructs and engineered blood vessels with diameters ranging between millimeters and hundreds of micrometers.^16−18^ However, the limited printing speed coupled with the trade-off between high printing accuracy and reduced mechanical properties of bioinks constrains their applicability in the case of microbranched networks at a capillary-like scale (4–10 μm).^19^ Thus, engineered tissue constructs still lack a highly dense 3D capillary network. In addition, control over microvasculature, including its orientation features, is a crucial aspect to guarantee tissue-specific functionality. For instance, the capillary network present in the human cerebral cortex possesses a peculiar tortuous and randomly organized structure in order to contribute to relatively uniform tissue perfusion and oxygenation.^20^ In skeletal muscle tissue, capillaries rearrange in an oriented fashion with the microvascular units and, in turn, align with the myofibers upon muscle injury.^21^ In a different way, adipose tissue presents different aspects of capillary orientation due to its dynamic nature, adapting to changes in metabolic demands such as weight increase and loss, mirroring its role in nutrient uptake, lipid storage, and hormone signaling.^22^ Consequently, controlling the orientation of a microvascular bed is crucial for the biofabrication of tissue-specific 3D structures.

In this framework, researchers continue to explore strategies to overcome random microvascularization and pave the way for functional and complex tissue implants, possibly increasing their applicability in clinics. In this study, highly dense biomimetic capillary networks were developed within the core–shell hydrogel microfibers. Such microvascular structures displayed a preferred orientation and encouraged the ultimate endothelialization-like process at the core–shell inner interface. To this aim, a fully automated microfluidics-assisted coaxial rotary wet-spinning system (3D RoWS) has been employed as an attractive and fast-prototyping alternative to conventional extrusion-based 3DBP techniques. Sodium alginate was selected to trigger the immediate gelation of the shell compartment, while the biomimetic, soft fibrin core was loaded with pro-angiogenic-like cells such as mesenchymal stem cells (MSCs) and human umbilical vein endothelial cells (HUVECs) to promote the formation of an oriented microvascular bed. After a thorough characterization of the presented method, the 3D RoWS platform was validated for the *in vitro* fabrication of MSC/HUVEC-laden core–shell hydrogel fibers in terms of viability and metabolic activity. Then, CD31 expression and orientation of the obtained microvessel-like networks were also evaluated. Interestingly, we demonstrated the rapid formation of a highly branched capillary bed with functional expression of key endothelial markers and overall structural architecture. These findings advance the effort to overcome the long-standing challenge of premicrovascularization in tissue engineering. Moreover, the core–shell design of the microfibers plays a crucial role in guiding and supporting the formation of the capillarized scaffolds. Finally, such fiber configuration can leverage the wide selection of biomimetic core materials aiming to create premicrovascularized, tissue-specific hydrogel constructs.

## 2 Results and Discussion

### 2.1 Microfluidics-Assisted Wet-Spinning Setup

**Figure 1:**
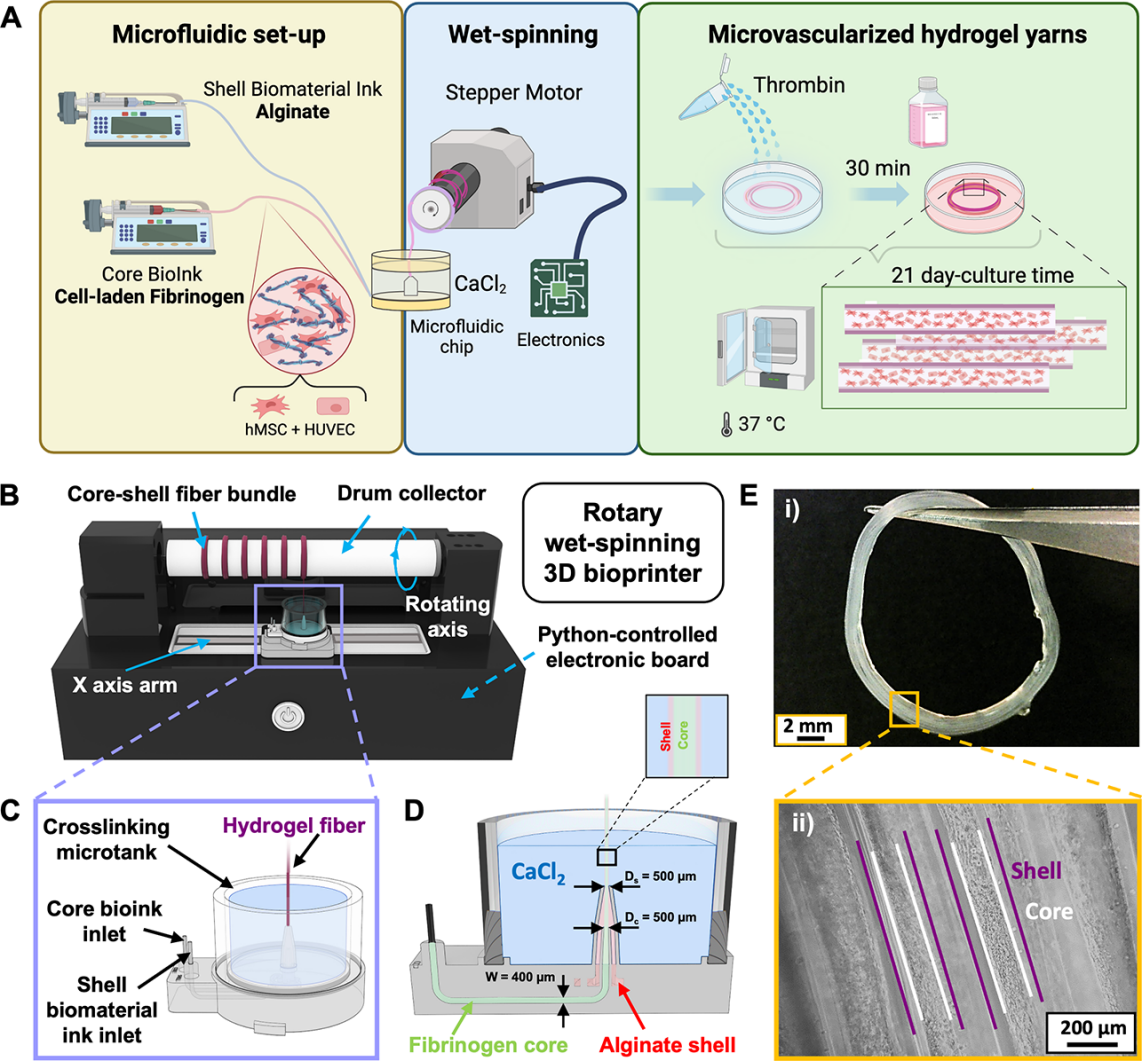
Setup of the automated 3D rotary wet-spinning biofabrication system (3D RoWS) for the production of microvascularized core–shell hydrogel microfibers. (A) Representative schematics of the overall experimental design. (B) 3D rendering of the 3D RoWS (KeyShot Pro 9.3 software, USA) continuously extruding a series of hydrogel bundles. (C) Overview of the microfluidic printing head (MPH) integrated into the microtank used for alginate cross-linking and fibrinogen prepolymerization. The two separate inlets enable the coupling of the prepolymer solutions loaded on the digital pumps and the simultaneous core–shell bioink delivery at the tip of the nozzle for the extrusion of microfibers. (D) Detailed design of the MPH equipped with the microtank used to fabricate the hydrogel yarns. The core and shell fluidic microchannels possess a squared diameter (*d* = 400 μm) and converge at the tip of the nozzle (⌀ = 500 μm) in the CaCl_2_-coagulation bath. (E) Wet-spun hydrogel bundle visualized as a (i) macroscopic image of the overall fibrous cell-laden scaffold (scale bar: 2 mm) and (ii) optical zoom-in of the highly aligned core–shell cellularized fibers. Scale bar: 200 μm.

In this work, a recently developed, unconventional 3D bioprinter for the microfluidics-assisted co-axial wet-spinning of hydrogel fibers^23,24^ has been employed for the biofabrication of microvascularized fibrous scaffolds (Figure 1A). The platform consists of an automated 3D rotary wet-spinning printer (3D RoWS) that relies on three main elements: (i) a microfluidic printing head (MPH) designed for the extrusion of core–shell fibers, (ii) a rotating drum collector (*D* = 25 mm, length = 180 mm) to gather the rapidly formed fiber, and (iii) an *X*-axis robotic arm (travel range = 160 mm) for the precise deposition of the extruded hydrogel thread onto the drum (Figure 1B). Notably, such an automated system enables the fiber-based thread number in each bundle to remain constant to continuously produce a series of fibrous scaffolds, while the MPH is laid out for the simultaneous extrusion of the alginate shell biomaterial ink and the fibrinogen core bioink (Figure 1C,D). More in detail, the MPH is designed with two inlets, dedicated to the delivery of the core- and shell-ink, respectively. Such inlets are microfluidically connected to the coaxial extrusion-based nozzle; thus, allowing the inks to be combined into a structured core–shell flow pattern at the tip of the nozzle. Remarkably, it is possible to finely control the number of overlapping threads to form a single bundle and the distance between adjacent bundles by tuning the 3D RoWS bioprinter parameters. Such advantageous aspect, together with the significant length of the drum collector and the *Y*-axis degree of freedom, enables the multiple biofabrication of fibrous scaffolds within the same wet-spinning session. The automated rotational motion of the drum, combined with the vertical orientation of the coaxial nozzle, leads to the formation of aligned fibrous scaffolds with marked anisotropic configurations (Figure 1E(i-ii)). Furthermore, the *X*-axis robotic arm of the 3D RoWS is designed to be slightly misaligned with respect to the drum collector, ensuring the MPH to be tangentially positioned with the drum and not directly placed under it. Such a configuration facilitates the fiber deposition at each drum rotation without disrupting the hydrogel.

The immediate ionic gelation of the alginate-based shell occurring within a coagulation microtank in the presence of calcium ions provides essential mechanical support during the wet-spinning session, as the fiber is gently and vertically pulled on the rotating drum from the coaxial nozzle. Moreover, while still wet from the ion-based cross-linking solution, the threads interact with each other, facilitating the bonding of alginate chains between nearby threads toward the creation of hydrogel-based fibrous scaffolds. Parallelly, the alginate shell enables the encapsulation of low-viscosity, cell-laden core bioinks that might otherwise be unsuitable for 3DBP due to the longer polymerization kinetics (e.g., enzymatic cross-linking).

Upon the reeling process, a secondary cross-linking was performed via an enzymatic-mediated reaction to stabilize the fibrin core. To this aim, a thrombin solution was added to the bundles to thoroughly gelify the fibrinogen fiber cores. Samples were incubated under cell culture conditions (37 °C, 5% CO_2_). Thanks to these features, such biofabrication system could be a potential platform for the creation of tissue-specific anisotropic architectures (e.g., muscle, tendon, nerve, and retinal tissues) with capillary-oriented microvascular beds that are generally difficult to obtain using conventional 3DBP approaches.

### 2.2 Characterization of Hydrogel-Based Materials

**Figure 2:**
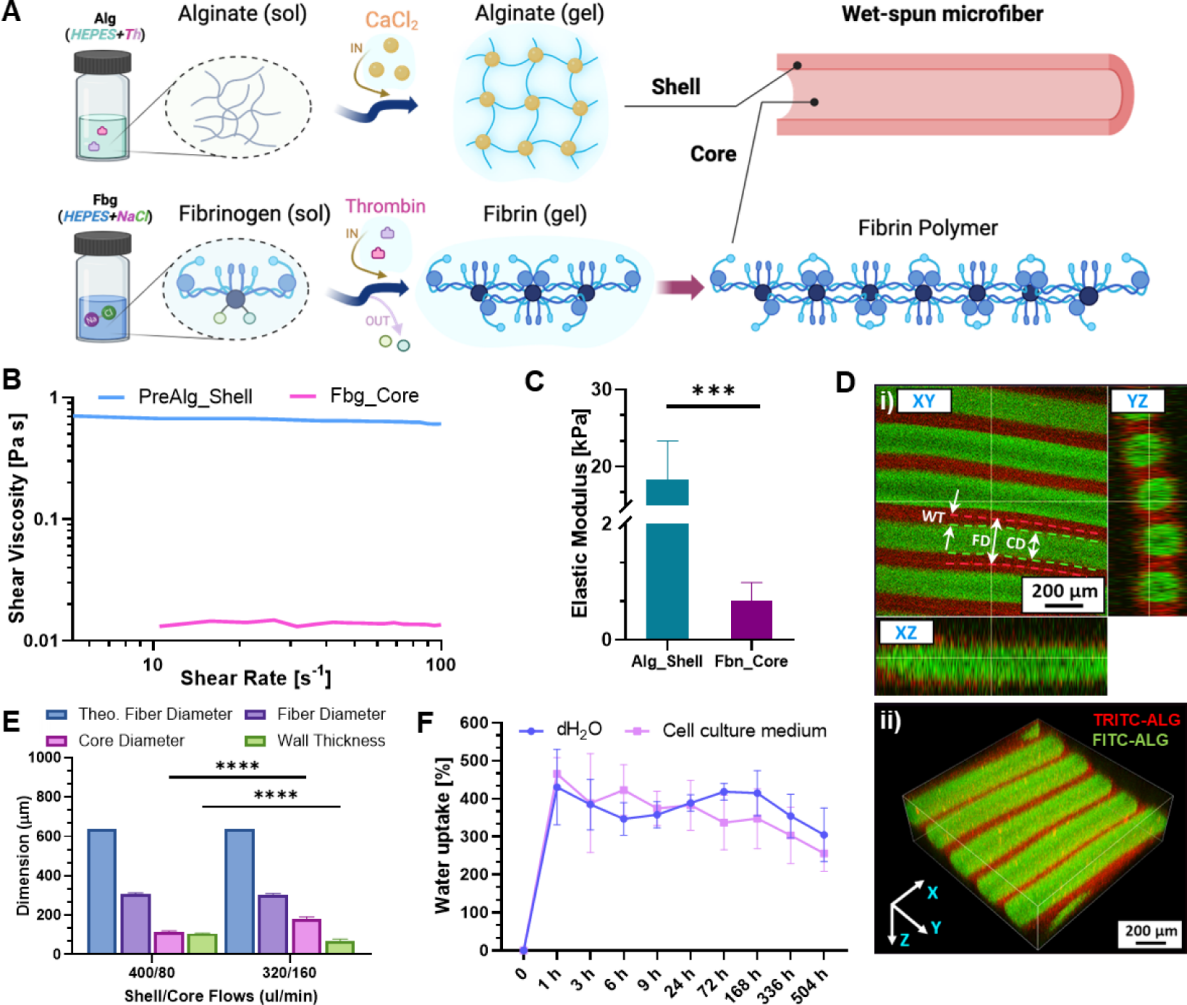
Characterization of the bioinks and fiber properties. (A) Schematics of the gelation mechanisms of 3% w/v alginate (shell (Alg)) and 1.4% w/v fibrinogen (core (Fbg)), which undergo ionic and enzymatic-mediated cross-linking in the presence of calcium chloride (CaCl_2_) and thrombin (Th), respectively. (B) Rheological properties of the prepolymer solutions in terms of viscosity *vs*. shear rate (PreAlg_Shell: alginate precursor, Fbg_Core: fibrin precursor (i.e., fibrinogen)). (C) Compressive elastic moduli of the alginate and fibrin hydrogels in terms of (Alg_Shell: alginate hydrogel, Fbn_Core: fibrin hydrogel) (*n* = 4). (D) Confocal images of a highly packed core–shell hydrogel-based fibrous bundle displaying the accurate and parallel arrangement of the fibers and the related well-defined core–shell compartmentalization on the different (i) XY-, YZ- and XZ-planes and as a (ii) 3D projection (FD: fiber diameter, CD: core diameter, and WT: wall thickness). Dashed lines highlight the whole FD (red) and CD (green). Scale bar: 200 μm. (E) Dimensional analysis of wet-spun fibers fabricated at different core–shell flow rates (total volumetric flow rate *Q* = *Q*_s_ + *Q*_c_: 480 μL/min) in terms of FD, CD, and WT compared to theoretical fiber diameter (*n* = 3). (F) Swelling properties of the fibrous scaffolds up to 21 days (shell: 320 μL/min, core: 160 μL/min, dH_2_O: deionized water, kept as a control) (*n* = 3). Significant differences: *p < 0.05, **p < 0.01, ***p < 0.001, and ****p < 0.0001.

Alginate and fibrin were, respectively, selected for the core–shell arrangement to (1) provide a stable sheath compartment able to precisely and immediately confine the inner phase and (2) tailor a soft cell-friendly core environment for the MSC/HUVEC coculture. The shell was formulated as an alginate-based solution containing ALG-HMW, ALG-LMW, and ALG-RGD at different concentrations. Specifically, ALG-HMW and ALG-LMW were mixed to balance the molecular weights in order to enhance the shell permeability, as well as the nutrient and oxygen diffusion for the biomimetic cell-laden core throughout the shell. In this view, the incorporation of RGD adhesive peptides via ALG-RGD addition was chosen to encourage cell adhesion and proliferation at the core–shell interface; thus, aiming at promoting the inner wall endothelialization of the core.^25^ Furthermore, the addition of thrombin into the shell prepolymer solution was designed to facilitate its diffusion at the core–shell interface during the wet-spinning process, thereby initiating the polymerization of fibrinogen to form fibrin and, in turn, ensure the alginate/fibrin interface stability. In fact, such a polymerization process is essential for the formation of a stable structure capable of supporting the encapsulated MSC/HUVEC cells (Figure 2A). More broadly, the alginate shell supplied a robust, easy-to-tune, and adaptable support for the compartmentalization of a soft core matrix hosting encapsulated cells, as a consequence of its immediate cross-linking occurring in the presence of CaCl_2_, where calcium ions (Ca^2+^) act as a cross-linking agent. Such interaction forms ionic bridges among alginate molecules, leading to the rapid gelation of the solution. The cross-linking mechanism can be controlled by changing the CaCl_2_ concentration, allowing for tuning both the mechanical properties and degradation rate of the alginate gel.^26^

Fibrinogen was chosen as a soft core biomaterial for its outstanding biocompatibility, minimal risk of adverse immune response, and ability to mimic the blood clotting mechanism, as it is a native component of blood plasma. Additionally, it plays a unique role in inducing angiogenesis, which is crucial for microvascularized tissue.^27^ The enzymatic reaction mediated by thrombin converts the soluble fibrinogen into insoluble fibrin strands, which polymerize and cross-link to form a soft 3D network. Based on our experience and current literature, we opted for an alginate solution of 3% (w/v) for the shell phase and a fibrinogen solution of 1.4% (w/v) for the core counterpart (see Section 4.2).^23^ The rheological properties of both precursor alginate hydrogel and fibrinogen solution showed a stable viscosity in the range of 10–100 s^–1^, thus showing a linear Newtonian-like behavior (Figures 2B and S1). Moreover, the marked difference in viscosity between the alginate and fibrinogen solutions (∼45-fold) highlighted the versatility of the 3D RoWS system in handling bioinks with a wide range of rheological properties. To further investigate the properties of the proposed hydrogels, their Elastic Modulus was evaluated using a compression test on cross-linked hydrogel bulks. It was observed that fibrin hydrogels exhibited a Young’s modulus of 0.67 ± 0.30 kPa. Such values are similar to the stiffness of soft-engineered ECMs that were previously found to support capillary-like network formation (0.1–1 kPa)^28,29^; thus, falling within the range of the microvascular endothelial stiffness of, e.g., lung tissue and vascular basement membrane (≤1.5 kPa).^30^ On the other hand, alginate constructs displayed significant 10-fold higher values (18.72 ± 4.88 kPa) as a supporting material for the wet-spinning of hydrogel-based core–shell microfibers (Figure 2C).^30,31^

### 2.3 Optimization of Wet-Spinning Parameters and Fiber Compartmentalization Characterization

The selection of flow rates for both the core and shell phases significantly influences the resulting fiber geometry and, in turn, can also affect the structural and functional properties of the constructs. The stability of the core–shell pattern was investigated in our previous work, highlighting the key role of volumetric flow rates in maintaining a stable core–shell interface, whereas the difference in flow rates between the core (*Q*_c_) and shell (*Q*_s_) phases generates a shear stress that stabilizes the pattern.^23^ Specifically, the volumetric flow rates selected for both core and shell phases lead to a shear stress that stabilizes the flow pattern at the core–shell interface for *Q*_s_ > 300 μL/min. In our study, two different sets of flow rates were further tested, avoiding excessive values and, in turn, shear stresses that could potentially damage cell survival within the core. In fact, this approach aimed at minimizing the shear stress while ensuring an efficient phase compartmentalization for the cell-laden constructs. The two sets were chosen to maintain the same total volumetric flow rate *Q* = *Q*_s_ + *Q*_c_ = 480 μL/min (i.e., *Q*_s_ = 400/*Q*_c_ = 80 μL/min and *Q*_s_ = 320/*Q*_c_ = 160 μL/min), while the rotation speed of the drum collector was set at 60 rpm (linear speed: 2π × drum radius × rpm = 79 mm/s). To understand the performance of the biofabricated structures, fluorescent-labeled bundles were wet-spun, and single fibers were analyzed in terms of fiber diameter (FD), core diameter (CD), and wall thickness (WT), and then compared to theoretical values of fiber diameter (TFD) (Figure 2D(i-ii)). Notably, the obtained FDs turned out to be lower than the TFD (i.e., TFD = 640 μm vs FD_400/80_ ∼ 309 μm vs FD_320/160_ ∼ 303 μm) in both cases (Figure 2E). Such results can likely be attributed to the shrinking effect of the alginate phase while exposed to Ca^2+^ and, to some extent, to the pulling force generated by the drum collector acting on the fiber.^32,33^ The size of FD in both sets did not statistically change (FD_400/80_: 309.34 ± 4.23 μm vs FD_320/160_: 303.76 ± 6.22 μm) as the total volumetric flow was kept constant. On the other hand, the CD (CD_400/80_: 116.37 ± 4.36 μm *vs*. CD_320/160_: 182.85 ± 9.08 μm) and WT (WT_400/80_: 107.25 ± 1.40 μm *vs*. WT_320/160_: 69.83 ± 8.72 μm) were significantly different. In fact, for small *Q*_c_ and large *Q*_s_, there is a contraction in the dimensions of the core phase. In contrast, a thicker core and a thinner shell were observed when *Q*_c_ is larger than *Q*_s_. In fact, particular attention was given to the WT dimension, which is crucial to properly ensure metabolic waste removal and nutrient exchange to the cell-laden core, whereas the diffusion limit of oxygen is limited to 100–200 μm to maintain cell viability,^34^ meaning that cells embedded within the core of the fiber must remain within this range from the fiber surface to maintain their viability. For this reason, the chosen set for subsequent cell experiments was Q_s_: 320/Q_c_: 160 μL/min (Figure S2A,B).

Nutrient diffusion, cell interaction, and mechanical stability are also influenced by the water absorption degree of the 3D constructs, which is essential for the formation of microvascular structures. Hence, optimized hydrogel bundles were also investigated in terms of swelling behavior in the cell culture medium (deionized water (dH_2_O) as a control). The results showed that the hydrogel-based structures exhibited a swelling degree of ∼465% in the cell culture medium within the first hour, reaching a plateau-like state on day 1. This rapid initial swelling occurs in the presence of a water concentration gradient between the external environment (i.e., the liquid buffer) and the hydrophilic polymeric network (i.e., the scaffolds). Here, water molecules quickly diffuse into the hydrogel, causing the polymer chains to absorb water and expand, thus enabling adequate water-like nutrient content for cellular functions. However, after 3 days, the liquid uptake of scaffolds started to decrease over the 3-week period time (cell culture medium ∼255% vs control ∼304% on day 21, respectively) (Figure 2F). This could potentially be due to the combined degradation process of the fibrin-based core as a consequence of its biochemical activity and low mechanical properties. Indeed, it is possible to speculate that both alginate and fibrin counterparts degraded at the same rate up to day 3 (61.57 ± 4.81%). Afterward, a noteworthy difference was detected up to day 21, highlighting the increasing role of fibrin degradation over the plateaued-like shell (Figure S3, Table S1).^35^ Such a trend could likely be attributed to the fibrinogen counterpart, which consistently swelled in cell culture medium and underwent hydrolytic degradation in its hydrogel state (i.e., fibrin). Also, the enzymatic activity of fibrinogen could influence its biodegradation in a physiological-like environment, due to the involvement of specific enzymes such as plasmin, which plays a crucial role in fibrinolysis.^36^ Notably, in the frame of fibrinogen-containing hydrogels, plasmin could catalyze the degradation of fibrin, leading to the gradual breakdown of the hydrogel structure.^37^ Accordingly, it is possible to speculate that the overtime degradation of the fibrin core could potentially lead to the formation of voids within the fibers, which may facilitate the development of capillary-like structures and enable cell migration.

### 2.4 Cell Studies

#### 2.4.1 Cell Growth and Proliferation Assessment of Cell-Laden Microfibers

**Figure 3:**
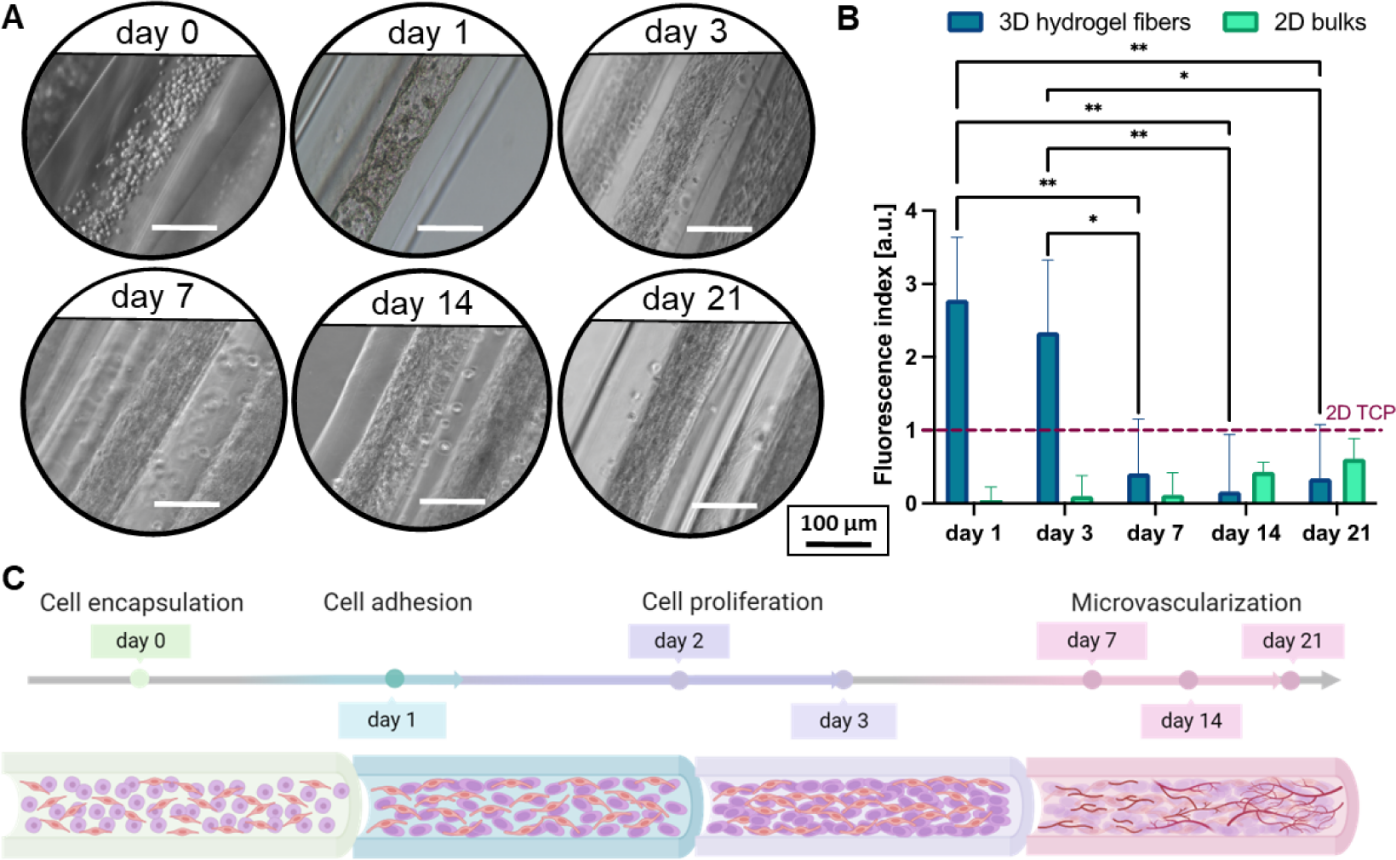
Biological response of MSC/HUVEC-laden hydrogels over a 3-week culture time period. (A) Representative optical images of the MSC/HUVEC population dynamics within the hydrogel fibers. Scale bar: 100 μm. (B) Cell proliferation of MSC/HUVEC in 3D fibrous scaffolds compared to 2D bulks and normalized to cells seeded on tissue culture plates (2D TCP, dashed line) (*n* = 5). (C) Schematics of the cell–material interaction occurring within the fibrin core upon cell encapsulation. Significant differences: *p < 0.05, **p < 0.01, ***p < 0.001, and ****p < 0.0001.

To demonstrate the compatibility of the proposed 3D RoWS for cell studies, the dynamics of cell attachment, cell growth, and proliferation of the MSC/HUVEC population encapsulated within the fibers was evaluated for up to 21 days. Initially, the coculture of MSC/HUVEC (2:1 ratio) was encapsulated within the core of the hydrogel fibers (see Section 4.8), where they were uniformly distributed in the soft fibrin matrix. Here, the low thrombin concentration employed to polymerize fibrinogen (i.e., 2 IU/ml) allowed for the formation of a homogeneous fibrin network able to support appropriate cell proliferation and spreading as a result of the prolonged enzymatic-mediated cross-linking time (∼30 min).^38^ Over time, the cells underwent significant growth, adapting and colonizing the confined fibrin-based core structure. At day 0, the encapsulated cells exhibited a predominantly round morphology, reflecting the early-stage encapsulation within the microfibers. Remarkably, a rapid transition was observed postencapsulation by day 1, as the MSC/HUVEC population actively initiated the formation of a primitive network (Figure 3A). This early cellular organization considerably progressed by day 3, suggesting a prompt adaptation within the fiber and a fast remodeling of the soft fibrin core environment. Although subsequent analysis up to day 21 revealed continued cellular growth, the proliferation rate did not display statistically significant differences post day 3, indicating a stabilization phase in the scaffold colonization process (Figure 3B). This could likely be attributed to two potential mechanisms. First, the confined core space of the hydrogel fibers may play a crucial role. Reasonably, available niches in the 3D network could become increasingly scarce over time, and, in turn, the compartmentalized fiber architecture limited cell division and proliferation. This was also confirmed by the increased degradation of the fibrin core after day 3 (see Section 1.2.2 in the SI). Second, MSC may have initiated a differentiation pathway after day 3 as a consequence of the interplay with HUVECs and the favorable fibrin matrix that could lead to cellular angiogenic differentiation.^39^ In fact, MSCs possess the capacity to differentiate into different lineages, including endothelial cells, and this can induce the formation of an early stage (pre-)microvascular network (Figure 3C, Section 2.4.4).^40^ Finally, such a pathway could have also been influenced by the cell culture medium, which was enriched in HUVEC supplements to support angiogenic differentiation (see Section 4.8). As a control, we also performed 2D cell coculture tests with the same cell ratio. Interestingly, cells cultured on 2D systems showed a lower proliferation rate compared to cells in the 3D fibrous scaffolds. This is likely due to the different environment given by the 2D culture, where cells are forced to adapt to a flat surface that neither mimics nor closely recapitulates a native tissue network, driving different cellular responses.^41^

#### 2.4.2 Microstructural Evaluation of Cell-Laden Microfibers

**Figure 4:**
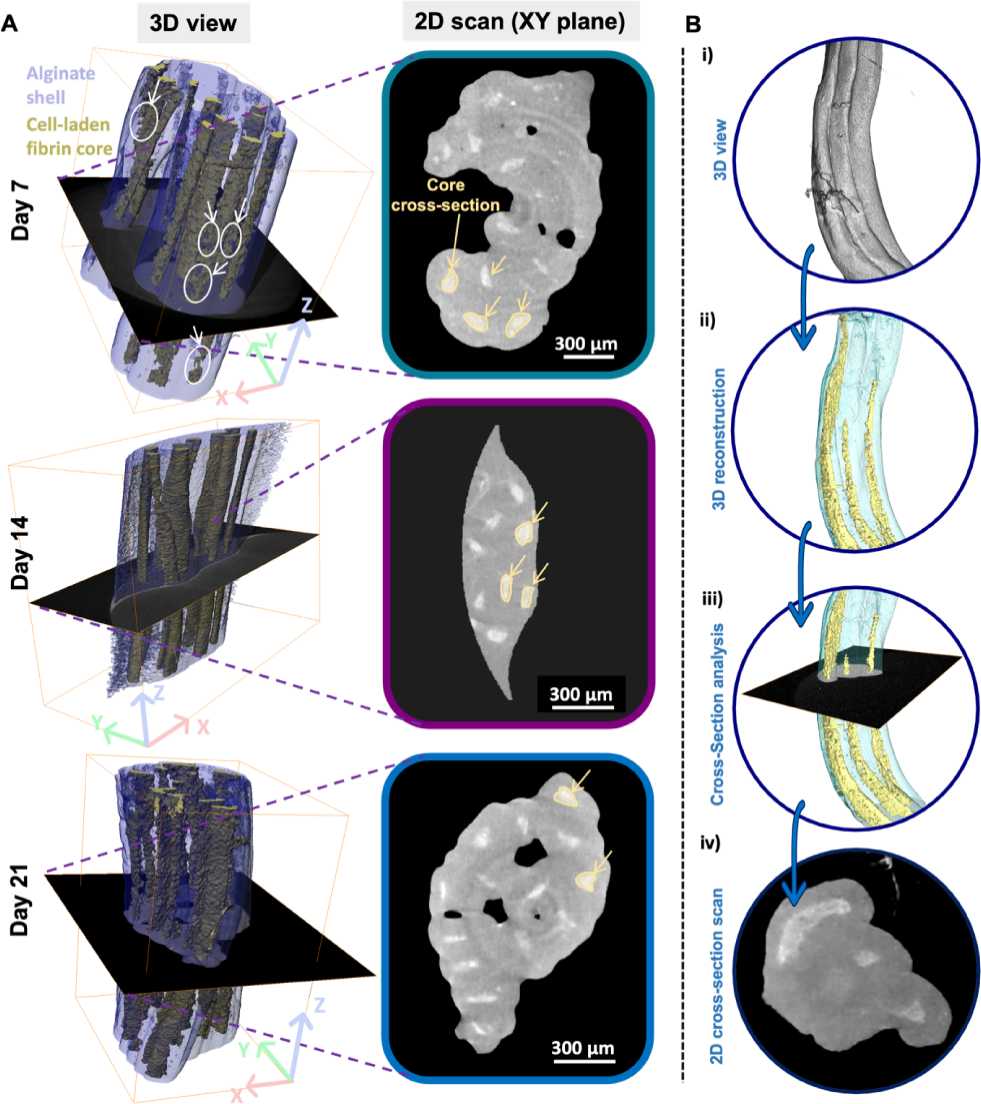
Microstructural evaluation of the cell-laden bundles via microcomputed tomography (μCT) scan reconstruction. (A) μCT-based 3D scan reconstruction and 2D view of MSC/HUVEC-laden bundles at day 7, 14, and 21 of culture. Legend - 3D view: white circles point to regions of interest with cell voids due to the ongoing proliferation process. Legend - 2D scan: yellow circles depict the core cross-section and related deformation due to fibrin softness. Scale bar: 300 μm. (B) Step-guided μCT reconstruction process of the cell-laden hydrogel fiber bundles distinguishing the core–shell fibers. The process led to obtaining a (i) 3D scan of the overall construct and its (ii) reconstruction, which allowed us to distinguish the two regions of each fiber (i.e., core and shell). Then, (iii) 2D plan selections and (iv) scans were obtained for a cross-sectional view of the fibrous constructs.

The fibrous scaffolds were microstructurally investigated to evaluate the spatial distribution of the cellular network within the fibrin core of the fibers. The 3D images reconstructed from the microcomputed tomography (μCT) confirmed a volumetric distribution of the MSC/HUVEC population over the 3-week culture period (Figure 4), showing a spatially homogeneous cell-laden core at days 7, 14, and 21. Consistently, these findings are in agreement with the cell proliferation results (Figure 3C), which revealed a plateau-like phase around day 3 with no further consistent proliferation after day 7 (Figure 4A). From the 2D cross-section scans, it was also possible to observe that the core compartment of the fibers tends to deviate from the theoretical circular shape given by the nozzle configuration. Such core deformation can be attributed to the inherent soft nature of the fibrin bioink, which also possesses relatively low viscosity (see Section 2.2). Furthermore, this dynamic might be especially stressed at reduced biopolymer concentrations and in the presence of encapsulated cells. In fact, these can exert forces on the surrounding matrix as they proliferate and migrate; thus, contributing to further volumetric deformation. In particular, MSC are known for their ability to remodel the ECM.^42^ To support these findings, the μCT volumetric reconstructions allowed us to distinguish and analyze the core diameter of the single fibers along the culture time (Figure S4), showing a core deformation between day 7 and day 21 (85 ± 5 μm *vs*. 55 ± 5 μm, respectively). Parallelly, they clearly revealed that some void spaces (i.e., cell-free fibrin) were still available within the core at early culturing stages. Such niches tend to be replaced by proliferated MSC/HUVEC cells at 2- and 3-week time points, as demonstrated by the 3D scans of the bundles. Overall, the fibers maintained their 3D structure, which was densely populated by cells, showing a uniform cell arrangement and spontaneous organization of the encapsulated MSC/HUVEC cells within the fibrin environment. Here, the core–shell configuration facilitated the confinement of the fibrinogen-based core, which may otherwise have been unsuitable with a conventional 3D bioprinting process. The distinct separation of phases was also detected by reconstructing the μCT volumes (Figure 4B) and by FIB-SEM imaging (Figure S5).

#### 2.4.3 Morphological Analysis of the Cell-Laden Bundles

**Figure 5:**
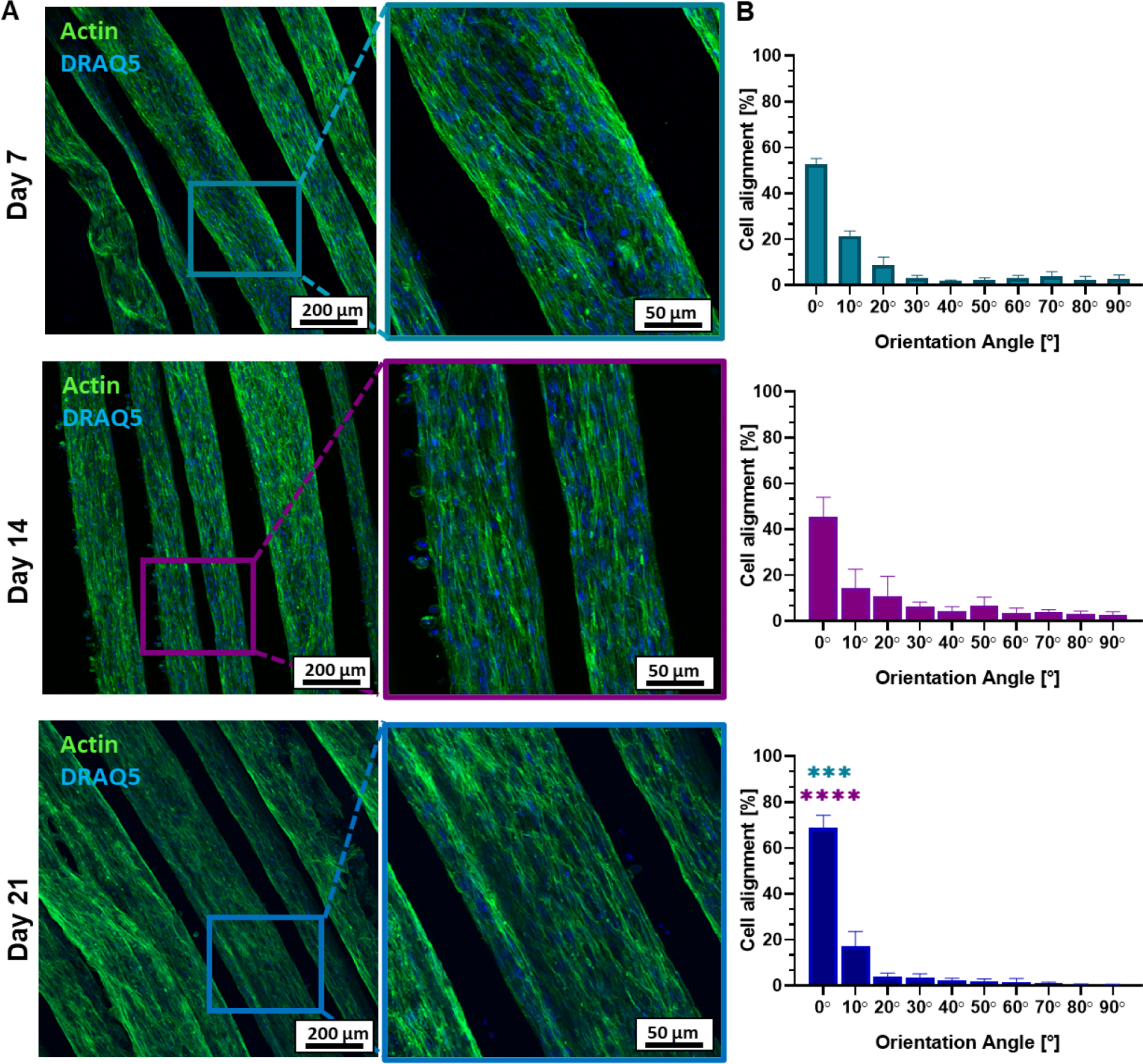
Morphology and alignment of MSC/HUVEC cultured within the core–shell hydrogel bundles. (A) Representative confocal images of actin (green, cell cytoskeleton) and DRAQ5 (blue, cell nuclei) on days 7, 14, and 21, displaying a preferential orientation of the cells along the longitudinal fiber axis. Scale bar: 200 μm (zoom-in: 50 μm). (B) Quantification of cell alignment over the respective time points (*n* = 3). The direction of the fiber axis is set equal to 0°. Significant differences: *p < 0.05, **p < 0.01, ***p < 0.001, and ****p < 0.0001.

Cell cytoskeleton morphology and cell alignment were studied at 1-, 2-, and 3-week time points (i.e., days 7, 14, and 21, respectively) upon biofabrication. The confocal images showed that MSC/HUVEC cells exhibited a well-spread morphology over the whole culture time, indicating robust cell growth within the fibrin matrix (Figure 5A). Overall, this evidence supported the successful coculture design of the study, thus showing the potential synergistic effect of both cell types.^43,44^ The elongated cellular shape was already evident on day 7, suggesting that the hydrogel environment promoted cell adhesion and spreading. By day 14, cells maintained their spread morphology, and the actin network indicated a favorable interaction between the heterogeneous cell population and the mature fibrin matrix. After 21 days, the cell population kept the spread morphology within the whole fiber’s core, suggesting a positive cell–material interaction in the proposed *in vitro* system. Taken together, such results could be attributed to the heterogeneous cell–cell interaction housed in the fibrin network, whose bioactivity, porosity, and biodegradation likely contributed to enabling a proper nutrient exchange and waste removal necessary for both cell expansion and ECM deposition, as also confirmed by the pH stability in the optimized cell culture medium (Figure S6).

Interestingly, the evidenced morphological behavior could also be influenced by the filopodia formation protruding from the actin network, which could sense the fibrous structure of the scaffolds and direct cell movement along the fiber axis (i.e., longitudinal direction, which was set as 0°).^45^ This was confirmed by the quantification of cell orientation, which marked a predominant cell alignment already detectable at day 7 and day 14 (0°: 52.79 ± 1.97% *vs*. 45.44 ± 7.05%, respectively) (Figure 5B). Notably, the longitudinal orientation of MSC/HUVEC at day 21 significantly increased (0°: 68.78 ± 4.52%) compared to both day 7 and day 14. Such results are in agreement with a previous study, which found a correlation between the fiber-based orientation of the fibrin hydrogel matrix and the maturation of the encapsulated cells along the fiber axis.^46^ In this framework, the fiber-based structure of the cell-laden hydrogel bundles facilitated an oriented cell attachment and distribution, whereas the wet-spinning biofabrication process might have positively affected the orientation of the biopolymer chains at a molecular scale.^47^ At a cellular level, the enhanced response of MSC/HUVEC integrin-mediated focal adhesions and cytoskeleton remodeling within the friendly ECM-like fiber core could also be boosted by the fiber orientation.^48,49^ Collectively, these findings validate the 3D RoWS system as a potential and effective technology for the production of highly aligned actin networks within the microfiber core.

#### 2.4.4 Microvascularization of the Cell-Laden Fibrous Bundles

**Figure 6:**
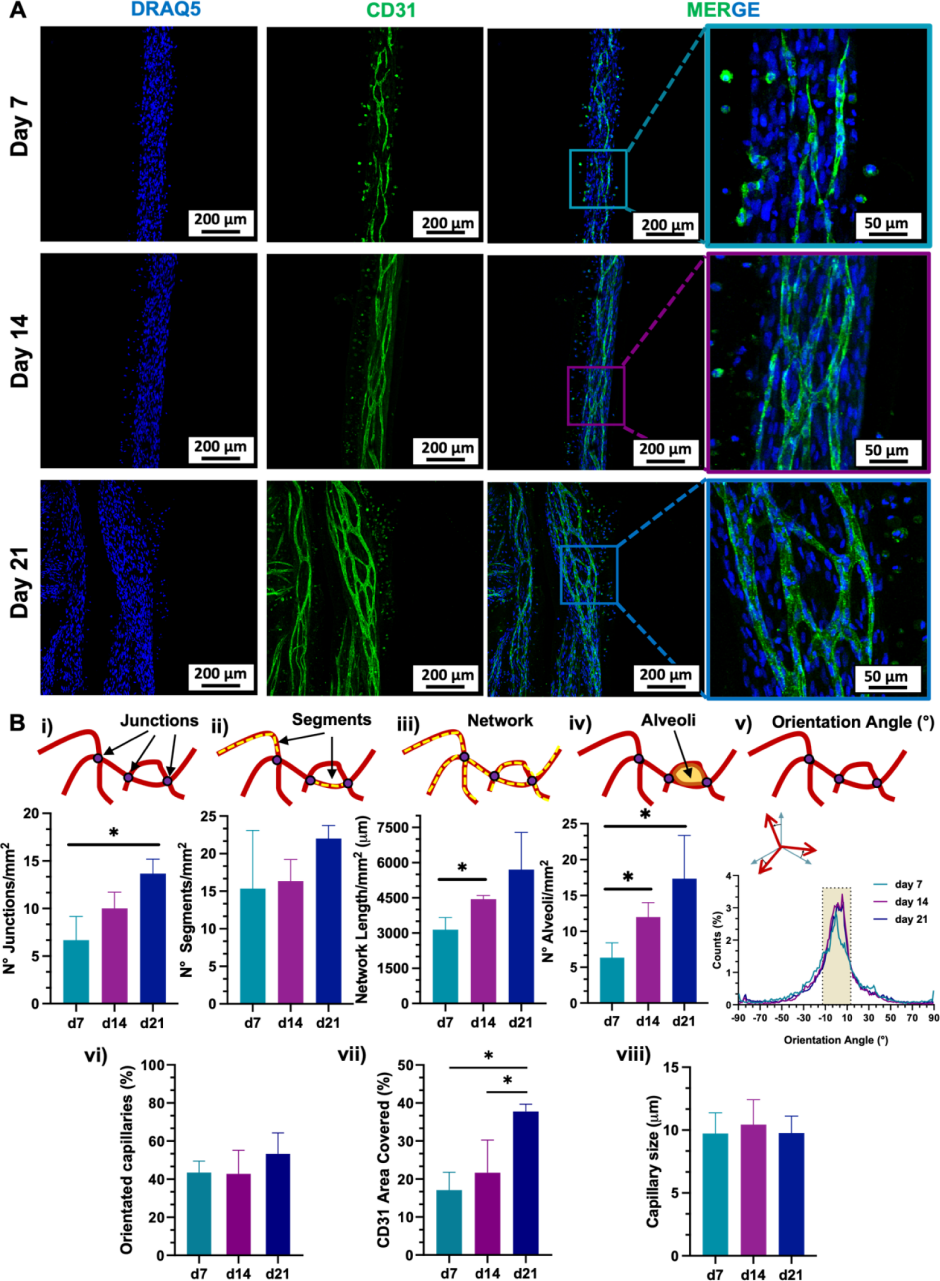
CD31 marker expression of MSC/HUVEC population encapsulated in the core–shell hydrogel bundles over a 3-week culture time period. (A) Representative confocal images of DRAQ5 (blue, cell nuclei) and CD31 (green, endothelial-like cell features) immunostained at day 7, 14, and 21, showing an interconnected capillary-like network oriented along the fiber axis and branching over the 3-week culture period. Scale bar: 200 μm (zoom-in: 50 μm). (B) Quantification of the capillary-like network on days 7, 14, and 21 (*n* = 3). CD31^+^ network characterization in terms of (i) junctions, (ii) segments, (iii) network length, and (iv) alveoli. The network orientation was profiled regarding (v) the distribution referred to orientation angles and (vi) the orientation along the fiber axis [pixel density within the angle range −10°, +10°], with the direction of the fiber axis set equal to 0°. (vii) Total area covered. (viii) CD31^+^ capillary size (*n* = 20). Significant differences: *p < 0.05, **p < 0.01, ***p < 0.001, and ****p < 0.0001.

Immunocytochemistry (ICC) analysis was conducted to evaluate the capability of the MSC/HUVEC coculture in promoting the maturation process of *in vitro* microvascular tissue, thus investigating the early endothelialization potential of the 3D microfibers in the presence of pro-angiogenic cells (Figure 6). This was assessed by staining the constructs with an anti-CD31 antibody (also known as platelet endothelial cell adhesion molecule-1 (PECAM-1)), as CD31 is a highly specific membrane protein expressed on the surface of functional endothelial cells and it is commonly used as a biomarker for intercellular junctions.

Confocal images of the cell-laden microfibers were taken over the 3-week culture time of the constructs (Figure 6A). On day 7, there was already evidence of CD31-positive (CD31^+^) cells, which denoted the early formation of capillary-like structures within the fibers. After 14 days, CD31^+^ cells started to organize a complex network whose density further intensified by day 21. This trend was also supported by quantitative analysis performed on the branched capillary network, which was thoroughly characterized in terms of junctions, segments, total length, and closed alveoli (Figure 6B). The number of capillary junctions in single fibers increased over time with a significant difference between day 7 and day 21 (d7: 6.67 ± 2.05 *vs*. d14: 10.00 ± 1.41 *vs*. d21: 13.67 ± 1.25, respectively) (Figure 6B(i)). This trend suggested an enhanced microvascularization within the hydrogel fibers as a result of the interconnected self-organized capillary network formation.^50,51^ However, the culture time did not influence the further development of the branching segments (Figure 6B(ii)). On the other hand, the increase in CD31^+^ network density was confirmed in terms of network length and alveoli’s number (Figure 6B(iii-iv)). Here, the complexity of the CD31^+^ network formation within a single fiber showed a significant increase between day 7 and day 14, expanding the total capillary length up to day 21 (d7: 3143.17 ± 424.53 μm *vs*. d14: 4441.43 ± 130.02 μm *vs*. d21: 5700.99 ± 1299.79 μm). To further characterize the architecture of the *in vitro* formed CD31^+^ capillary bed, the orientation of the microvasculature was evaluated considering the angle range ±10°, where 0° was set as the direction of the microfiber longitudinal axis. Remarkably, the distribution of the capillary network was found to be oriented along the longitudinal direction of the fiber over the whole culture time (Figure 6B(v)). Parallelly, the percentage of oriented capillaries in the target angle range (−10°, +10°) increased after a 2-week period (d7: 43.47 ± 4.92% *vs*. d14: 42.75 ± 10.12% *vs*. d21: 53.31 ± 8.94%, respectively) (Figure 6B(vi)). Furthermore, the quantification of the fiber area covered by CD31^+^ cells within the constructs confirmed the overall maturation of the capillary network over the culture time, which in turn maintained their ability to closely mimic the native size of capillary blood vessels, typically ranging from 4 to 10 μm (Figure 6B(vii-viii)).^3^ This *in vitro* recapitulation of physiological capillary dimensions (d7: 9.73 ± 1.66 μm, d14: 10.44 ± 1.98 μm, and d21: 9.76 ± 1.36 μm, respectively), which can also slightly vary depending on the specific tissue and function (e.g., capillary bed: 10–15 μm),^52^ is critical for supporting effective nutrient and oxygen exchange and ensuring appropriate microvascularization at the local level. Moreover, it did not show any significant size change during the 21-day culture period. Taken together, such findings suggested that both the soft fibrin matrix and fiber alignment had a potential role in CD31^+^ cell adhesion, inducing the formation of an oriented and interconnected capillary network.^53^ At the cellular level, MSCs can indirectly enhance the tissue maturation and the angiogenic capacity of the constructs by secreting a wide range of growth factors, such as vascular endothelial growth factor (VEGF), FGF, and transforming growth factor-beta (TGF-β), that can stimulate the proliferation and migration of endothelial cells.^54,55^ Moreover, the capillary network orientation might also be attributed to the MSC integrin-mediated cell–cell signaling, which likely led to the secretion and upregulation of angiogenic proteins within the engineered constructs.^56,57^ Parallelly, HUVECs could boost the pro-angiogenic properties of MSCs through direct cell-to-cell contact, promoting the formation of capillary-like structures and potentially influencing their differentiation pathways, as confirmed by the limited proliferation rate detected after 3 days of culture. Conversely, the MSCs’ angiogenic pathway can enhance the interaction with HUVECs in the presence of an ECM-enriched microenvironment suitable for angiogenesis and capillary alignment.^58−60^ For instance, it was recently demonstrated that hydrogel-embedded MSC/HUVEC population increased the expression of cell–cell junction proteins, secretion of angiogenic factors, and proliferation and migration of HUVEC, ultimately leading to the formation of microcapillaries within the hydrogels.^61^ Thus, the proposed 3D coculture system can be a powerful tool in enhancing both angiogenesis and tissue integration by providing MSC differentiation and improving the formation of vessel-like structures.^62^

**Figure 7:**
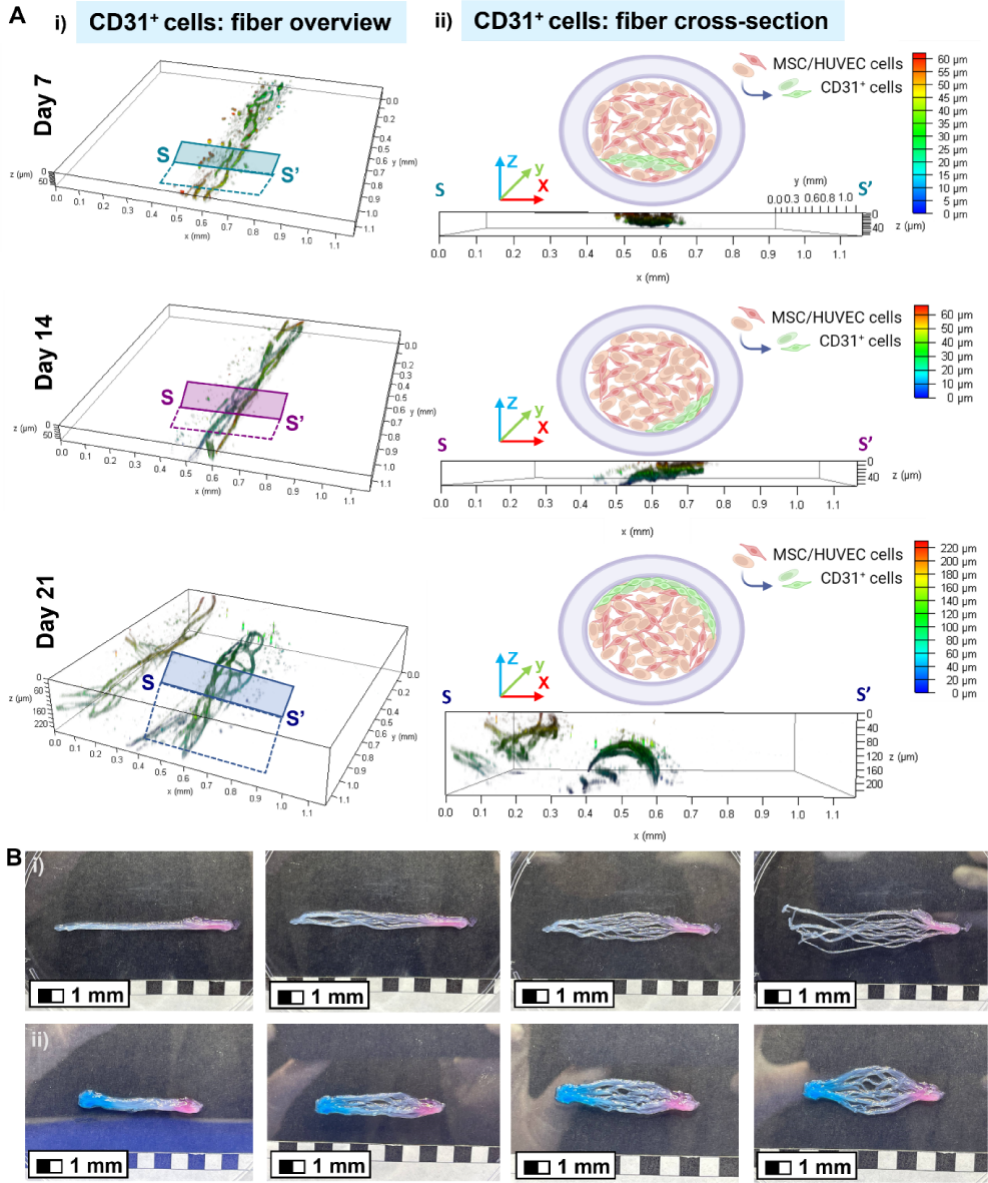
Distribution of the CD31^+^ MSC/HUVEC population within the core of the hydrogel microfibers bundles and the microvascularization potential of the fibrous scaffolds. (A) 3D confocal renderings of interconnected CD31^+^ cells immunostained on days 7, 14, and 21 obtained from representative confocal images. The color maps show the Z-depth and location of the expressed marker within the fibrin core. XY area = 1.1 × 1.1 mm^2^. (i) Fiber overview and (ii) fiber cross-section (XZ-plane) are depicted together with a simplified illustrative sketch displaying the endothelialization-like maturation toward the inner wall of the core (green cells: distribution overview of CD31^+^ cells depicted in (A)). (B) Proof-of-concept of the microvascularization potential of the hydrogel-based fibrous scaffolds, which can be adapted to a target configuration designed for the implantation site at specific anatomical areas. The wet-spun ring bundles were cut to obtain yarns customizable in terms of overall length and fiber density. Alginate droplets were pipetted on (i) one or (ii) both extremities to simulate a bioadhesive fixation at the implantation site. For illustrative purposes, red and blue food-grade dyes were added to distinguish hypothetical oxygenated and deoxygenated blood capillaries, respectively. (i) Microvascularization for the capillary-like development of a fan-shaped bed and (ii) a closed branched system. Scale bar: 1 mm.

Interestingly, the 3D renderings obtained from the projection of confocal images revealed that the core–shell fibers underwent a more pronounced CD31^+^ network-based endothelialization-like process in proximity of the inner wall of the fibers’ core (Figure 7). Here, it was possible to identify a volumetric capillary network distribution featuring interconnected structures that link different levels of the fiber layers at different time points (Figure 7A(i-ii)). This aspect led to speculate on a crucial microvascularization outcome of the proposed engineered constructs. In particular, it was found that CD31^+^ cells were subject to a dynamic spatial change after 7 days of culture. Indeed, cells likely started to migrate toward the inner wall of the core phase, thus establishing a surface endothelialization-like process as depicted on day 14 and pronouncedly evidenced on day 21, respectively. This aspect is in line with a recent work that demonstrated the capability of low-viscosity bioinks for the microfluidic 3DBP of endothelial cell-laden hydrogel fibers, promoting cell migration and alignment.^53^ Such findings highlight the potential of the 3D RoWS for creating complex microvascular trees, especially within anisotropic environments.^63,64^ Moreover, by tuning the design of the MPH used for fiber extrusion, one may achieve fiber dimensions that can mimic the native diameters of anatomically different types of blood vessels (e.g., capillaries, arterioles, venules, etc.; Figure S7). In fact, the hydrogel-based scaffolds could be spatially tuned to fit the target configuration for a potential implantation site. By cutting such fibrous ring bundles, both the length and fiber density of the yarn can be customized to match the specific needs of the area. Such flexibility ensures precise adaptation to the anatomical requirements (Figure 7B(i-ii)).

Collectively, the 3D RoWS can be considered a technology suitable for the biofabrication of fiber-based tissue-engineered constructs aimed at providing an oriented capillary-like network for microvascularized structures. Here, the interaction between MSCs and HUVECs played a significant role in promoting an oriented fiber capillarization, highlighting the capability of the system to potentially *in vitro* prevascularize different engineered tissues, such as skeletal muscle, where the orientation of native blood capillary vessels is intricately linked to the tissue-specific functions and metabolic requirements. Besides, the successful formation of fiber-based oriented capillary-like networks could envision an efficient nutrient exchange and waste removal throughout highly complex 3D fibrous scaffolds. In light of this, our study unveils certain limitations that should be addressed in future research. First, the tracking of MSC and HUVEC interactions within the constructs could be explored further to deepen our understanding of the underlying co-culture dynamics. Second, although this study demonstrated the successful *in vitro* formation of capillary-like networks, the remaining challenges include exploring the dynamic compliance, the perfusability, and the wall thickness of the proposed capillary-like structures with an improved configuration of the setup. Finally, the validation of their functionality and integration *in vivo* would open a new pipeline to translate these engineered constructs from the bench to bedside.

## 3 Conclusions

In this study, we demonstrate the convergence of extrusion-based 3D bioprinting and vertical microfluidics-assisted wet-spinning techniques to rapidly produce highly aligned hydrogel-based bundles for the recapitulation of an oriented CD31^+^ microvascular-like network. By decoupling the bioink’s rheological properties from the printing accuracy, we successfully wet-spun low-viscosity bioinks into compartmentalized core–shell fibers.

The heterogeneous MSC/HUVEC coculture encapsulated in the soft fibrin core rapidly proliferated and uniaxially aligned *in vitro* over a 3-week period, forming a branched capillary network similar to a microvascular bed. This aspect was further confirmed by the expression of specific endothelial markers (CD31), mimicking the morphological complexity of anisotropic microvascularized native tissues, such as skeletal muscles. In conclusion, these findings show great potential for significantly enhancing the multiscale biofabrication of biomimetic and oriented microvascularized tissue-specific engineered constructs, while also envisioning the applicability of the proposed platform in the framework of microvascular disease modeling.

## 4 Materials and Methods

### 4.1 Materials

All the materials, including Triton X-100, HEPES, human basic fibroblast growth factor (hbFGF), goat and bovine serum albumin (BSA), and fibrinogen (bovine plasma, Type I-S, 65–85% protein), were purchased from Merck, Germany (formerly Sigma-Aldrich, USA) and were not further purified unless otherwise stated. Low molecular weight sodium alginate (ALG-LMW, 33 kDa) and high molecular weight sodium alginate (ALG-HMW, 100 kDa) were kindly gifted by FMC Biopolymers (USA). Alginate-RGD was synthesized in-house as reported in the Supporting Information. Calcium chloride (CaCl_2_) and sodium chloride (NaCl) were purchased from Eurochem BGD (Poland). Minimum essential medium alpha (α-MEM), fetal bovine serum (FBS), trypsin-EDTA, penicillin-streptomycin, Alexa Fluor 488 Phalloidin, Alexa Fluor 488 goat antirabbit secondary antibody, and DRAQ5 were purchased from Gibco Invitrogen (USA). Antirabbit polyclonal CD31 antibody was purchased from Abcam (United Kingdom). 4-(2-Hydroxyethyl)-1-piperazineethanesulfonic acid (HEPES) was purchased from Roth GmbH (Germany). Human bone marrow-derived mesenchymal stem cells (MSCs), human umbilical vein endothelial cells (HUVECs), and endothelial cell growth medium (EGM-2 Cell Growth Medium BulletKit) were purchased from Lonza (Switzerland).

### 4.2 Hydrogel-Based Ink Preparation

Core–shell hydrogel fibers were manufactured using two different hydrogel formulations for the shell and core compartments, respectively. The shell was composed of an alginate-based biomaterial ink for microfluidic wet extrusion, while the core was a soft cell-laden fibrin bioink.

#### 4.2.1 Shell Composition

Sodium alginate in its final formulation was obtained using a dual-stage method. First, alginate was prepared by dissolving 3.3% (w/v) alginate (2.2% (w/v) ALG-LMW + 0.55% (w/v) ALG-HMW + 0.55% (w/v) ALG-RGD) in HEPES solution (25 mM, pH 7.4). Afterward, this prepolymer formulation was sterile-filtered (0.22 μm), and thrombin (20 IU/ml) was added at a 1:10 v/v ratio (final: 2 IU/ml thrombin solution) to obtain the final formulation of 3% (w/v) alginate (2% (w/v) ALG-LMW + 0.5% (w/v) ALG-HMW + 0.5% (w/v) ALG-RGD).

#### 4.2.2 Core Composition

Fibrinogen was prepared at a final concentration of 1.4% (w/v). The powder was dissolved in HEPES-NaCl buffer (25 mM–0.15M, pH 7.4), gently stirring at a minimum speed to avoid undesired agglomerations and cluster formation. The final solution was sterile-filtered prior to use.

### 4.3 Wet-Spinning Setup

The wet-spinning platform consists of (1) a microfluidic printing head (MPH) coupled with a cross-linking microtank (volume (*V*) = 10 mL) housing a coagulation bath with a coaxial nozzle (inner needle ID = outer needle OD = 500 μm) to allow for the immediate cross-linking of the core–shell fibers, (2) a Teflon-based rotating drum collector (*D* = 25 mm and length = 180 mm), and (3) an extrusion *X*-axis track. The overall platform was controlled by an electronic board programmed in a Python environment, which allows the user to define the specific parameters of the 3D RoWS (e.g., rotational axis speed, X offset, and fibers-per-bundle ratio). The MPH was fluidically coupled with a dual digital pump (Cetoni GmbH, Germany) by means of polyethylene micro-medical tubings (ID = 0.8 mm) to let the inks flow toward the core and shell microchannels (squared, 400 × 400 μm), respectively. To this aim, the tubings were independently connected to the core and shell inlets of the MPH, previously placed on the *X*-axis motion arm of the platform. Then, the prepolymer solutions were loaded into the syringes and pushed toward the MPH at selected flow rates to automatically deliver the inks at constant flow rates, thus generating a coflow system at the tip of the extrusion nozzle.

Optimized standard volumetric flow rates for core (*Q*_c_) and shell (*Q*_s_) inks were set as *Q*_c_ = 160 μL/min and *Q*_s_ = 320 μL/min, respectively. The coagulation bath was filled with a 0.3 M CaCl_2_ solution to allow for the rapid gelation of the alginate shell at the tip of the nozzle, where an ionic reaction between Ca^2+^ ions and sodium alginate chains occurs and alginate immediately polymerizes.^64^ At this stage, the forming fiber was gently clamped by means of tweezers close to the tip of the nozzle and carefully placed on the Teflon-based rotating drum collector to continuously lay down for the serial collection of hydrogel bundles. The rotational speed of the drum was set at 60 rpm (i.e., 79 mm/s), and the number of threads was maintained at a constant figure (30 threads/bundle, 30 s of extrusion time) in each bundle. Afterward, the fabricated constructs were gently peeled off from the mandrel and immersed in thrombin/HEPES buffer solution (2 IU/mL −25 mM, pH 7.4) for 30 min under incubated conditions (37 °C, 5% CO_2_) to enzymatically polymerize the fibrinogen-based core. Subsequently, the buffer was removed, and the hydrogel fibrous scaffolds were cultured in a cell culture medium for up to 21 days.

### 4.4 Prepolymer Formulation Studies

#### 4.4.1 Rheological Properties

The prepolymer formulations were tested at 25 °C using a Brookfield DV-II+ Pro Rheometer (Brookfield, Massachusetts, USA), equipped with a HADV-II+Pro spindle. The viscometer speed was set at 100 rpm and then at 200 rpm. Shear viscosity was evaluated by applying shear rates ranging from 0.1 to 100 s^–1^.

### 4.5 Mechanical Testing

#### 4.5.1 Elastic Modulus

The compressive test on bulk hydrogels was performed (*n* = 4 per ink formulation) after 72-h incubation in dH_2_O to let them reach the swelling plateau, using a Dynamic Mechanical Analyzer (DMA Q800, TA Instruments). Mechanical tests were conducted at room temperature (RT) with a preload of 0.001 N, by applying a compressive strain ramp at 5% min^–1^ until 30% strain. From stress–strain (σ–ε) curves, the elastic modulus (*E*) was calculated as the slope in the linear 0–5% strain region (*R*^2^ > 0.9).

### 4.6 Fiber Characterization

Fiber constructs were produced and characterized in terms of both single fibers and fiber bundles. Single fibers were characterized in terms of wet spinnability and diameter. Bundles were investigated in terms of the swelling. To investigate the core–shell fibers, alginate conjugated with fluorescein isothiocyanate (FITC-ALG) was used to label the shell (5 mg/mL replacing ALG-HMW and ALG-LMW within the corresponding formulation, respectively), while tetramethylrhodamine-conjugated alginate (TRITC-ALG) was used to fluorescently label the core (1% w/v ALG-LMW, i.e., 0.5% w/v ALG-LMW + 0.5% w/v TRITC-ALG LMW). FITC/TRITC-ALG was synthesized in-house as reported in the Supporting Information.

#### 4.6.1 Spinnability and Fiber Diameter

Wet-spinning parameters were tuned to identify the optimal combination of both shell and core flow rates (i.e., *Q*_s_ and *Q*_c_, respectively) to produce highly compartmentalized core–shell microfibers spun with the proposed biomaterial ink formulations (see Section 1.2 in SI). Fluorescence images (Leica TCS SP8) of the core–shell fibers (core: TRITC-Alginate, shell: FITC-Alginate) were wet-spun considering two different volumetric flow couplings, while keeping constant the total flow rate set as *Q* = *Q*_s_ + *Q*_c_ = 480 μL/min, and were used to measure fiber parameters (i.e., fiber diameter (FD), core diameter (CD), and wall thickness (WT)). Three different positions of a single fiber were selected from three different bundles (*n* = 3) produced at the same speed rate (60 rpm). Wall thickness was calculated as WT = (FD – CD)/2. Theoretical fiber diameter (TFD) was calculated according to eq 1:
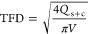
1

Where *Q*_s+c_ = *Q* = *Q*_s_ + *Q*_c_ is the total volumetric flow and *V* is the motor speed.^32^

#### 4.6.2 Swelling

The swelling test was performed in both coculture medium and dH_2_O (*n* = 5) (see Section 2.3). Fibrous bundles were produced as described in Section 4.3. Upon fabrication, the 3D constructs were immersed in a buffer of HEPES 25 mM CaCl_2_ 0.2 M. Afterward, constructs were soaked in a series of concentrated ethanol (50%, 70%, 90%, and 100%, 15 min each) and left to dry overnight. The dry mass was weighed (*M*_d_). Then, samples were immersed in the selected buffer supplemented with sodium azide (0.05% w/v) (POCH S.A., Poland) as a bacteriostatic agent. The structures were incubated at 37 °C (MaxQ 6000, Thermo Fisher Scientific, USA). Time points were set at 1, 3, 6, 9 h and 7, 14, and 21 days, respectively). At selected time points, all wet samples were gently swabbed, weighed (*M*_w_), and placed back in the buffer. The water content (*W*) was calculated by following eq 2.
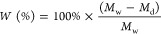
2

### 4.7 Microcomputed Tomography (μCT)

Cell-laden wet-spun hydrogel bundles were fixed at selected time points (i.e., days 7, 14, and 21, respectively), cut, and placed into 1.5 mL Eppendorf-type tubes to keep the samples from moving while scanning. The tubes were mounted in the device’s sample holder, contrasted with iodine solution, and scanned in oil immersion using a microfocused X-ray tomographic system (MICRO XCT-400, Xradia, Zeiss, Germany) with a 40 kV electron acceleration voltage and a 250 μA current. For each sample, 1000 projection images were recorded with a detector exposure time of 6 s in step mode at a 10× objective. The scans were performed with a spatial resolution of 2 × 2 × 2 μm in voxel size. The initial volume reconstruction was performed using the XMReconstruction software (Zeiss, Germany). Then, volumes were exported to Avizo Fire software (version 2020.2; FEI Visualization Sciences Group). Here, 2D planes and 3D reconstructions were obtained for a qualitative analysis.

### 4.8 Cell Studies

Human bone marrow-derived mesenchymal stem cells (MSCs) were expanded in α-MEM (Gibco, USA) supplemented with 10% FBS, 1% penicillin–streptomycin (10,000 U ml^–1^ penicillin, 10 μg mL^–1^ streptomycin; Gibco, USA), and 1 ng mL^–1^ human basic fibroblast growth factor 2. Human umbilical vein endothelial cells (HUVECs) were cultured in a dedicated medium (EGM-2 Endothelial Cell Growth Medium BulletKit, Lonza). Cells used for 3D RoWS were from passage 3 to 6. The optimized medium consisted of EGM-2 supplemented with α-MEM at a volume ratio of 4:1, which was changed every other day for the cultured constructs. For wet-spinning experiments, the MSC/HUVEC-laden bioink was prepared by mixing fibrinogen as a hydrogel precursor with HUVEC and MSC at a 1:2 ratio,^43^ respectively, to achieve a final density of 1.5 × 10^7^ cells mL^–1^. Prior to wet-spinning, both tubing and MPH were washed with 70% ethanol (EtOH) and then with sterile dH_2_O under aseptic conditions. Then, a UV cycle was run before connecting both ink-loaded syringes and digital pumps. The as-prepared bioink was loaded into a sterile glass syringe and then microfluidically coupled with the shell biomaterial ink, according to the chosen wet-spinning parameters (*Q*_s_ = 320 μL/min, *Q*_c_ = 160 μL/min, 60 rpm, continuous flow). Finally, the hydrogel bundles were wet-spun, removed from the mandrel, and cultured in the optimized medium for up to 21 days upon enzymatic polymerization of fibrinogen in thrombin solution.

#### 4.8.1 Cell Viability and Proliferation Assessment

##### 4.8.1.1 Proliferation Assay

PrestoBlue (Thermo Fisher Scientific, USA) was assessed in a 9:1 cell culture medium/PrestoBlue reagent solution. The solution (10% v/v PrestoBlue) was added to each sample of cell-laden fibers and 2D tissue culture plate (TCP) controls, seeded with the same cell density (*n* = 5). Specimens were divided into four replicates for each condition after 1, 3, 7, 14, and 21 days and incubated for 1 h (37 °C, 5% CO_2_) in agreement with the protocol. Herein, 100 μL from each well was transferred to a 96-well plate and analyzed with a plate reader (FLUOstar Omega, BMG Labtech, Germany), with an excitation wavelength at 530 nm and emission at 620 nm.

#### 4.8.2 Cell Distribution

The qualitative cell distribution within the fibrin core was evaluated by imaging the scaffolds in a bright field mode (Leica TCS SP8, Germany). At set time points, samples were imaged to investigate in detail the evolution of cell proliferation during the culture time of up to 21 days.

#### 4.8.3 Cell Morphology and CD31 Expression

##### 4.8.3.1 Actin/DRAQ5

Cell morphology was investigated by staining the cell actin filaments and nuclei. Scaffolds were fixed using 4% paraformaldehyde (v/v) for 30 min. Then, they were washed in HEPES 25 mM three times (5 min each). Subsequently, 0.3% (v/v) Triton X-100 prepared in HEPES was added for 15 min, and the samples were washed three times. Afterward, constructs were incubated in 1% (w/v) BSA in HEPES for 30 min to inhibit any nonspecific binding. Alexa Fluor 488 Phalloidin in HEPES (1:40) was added for 40 min at RT. Samples were washed and incubated in a 1:1000 DRAQ5 solution in HEPES for 10 min. Finally, the fibrous scaffolds were imaged with a confocal microscope (Leica Systems) to quantify cell alignment using ImageJ/Fiji (National Institute of Health (NIH), USA). Herein, three different images of three different scaffolds (*n* = 3) were analyzed by measuring the cytoskeleton orientation with respect to the fiber axis (ImageJ software, OrientationJ plugin).

##### 4.8.3.2 CD31 Immunostaining

After fixation at desired time points (i.e., days 7, 14, and 21), scaffolds were washed thrice in sterile HEPES 25 mM. To effectively enhance antibody penetration within the cell-laden core of the fibers, constructs were treated with 0.08% (w/v) alginase in saturated EDTA (pH 8) for 5 h at 37 °C (5% CO_2_) and washed in 25 mM HEPES buffer. Then, 0.3% (v/v) Triton X-100 in HEPES was used to permeabilize the constructs for 15 min. Afterward, an additional washing was performed, and then the bundles were blocked for 30 min in 1% (w/v) bovine serum albumin (BSA) prepared in HEPES buffer. Subsequently, an anti-CD31 primary antibody (Abcam 28 364, United Kingdom) was added to the scaffolds (diluted 1:20 in HEPES) and incubated overnight at 4 °C. Then, the constructs were washed three times, and the goat antirabbit secondary antibody was added (Alexa Fluor 488, 1:300 in HEPES) for 2 h at RT. Finally, the secondary antibody was removed, and DRAQ5 (1:1000 in HEPES) was added for 15 min to stain cell nuclei. Prior to imaging, the hydrogel bundles were HEPES-washed thrice.

#### 4.8.4 Microvascular Features Quantification and Orientation

To automatically calculate capillary metrics, the Angiogenesis Analyzer plugin of ImageJ/Fiji (NIH, USA) was used.^65,66^ A segment was defined from one branching point to another where a single CD31^+^ capillary branched into two or more vessels. The number of junctions was calculated as the number of intersections generated from a minimum of two CD31^+^ capillary-like segments expressed within each single fiber (*n* = 3). The total network length was calculated as the overall sum of each CD31^+^ segment detected on single fibers. The number of alveoli was defined as the total number of areas obtained by intersecting two different CD31^+^ segments on the same two junctions. The CD31^+^ capillary network orientation was analyzed by using the OrientationJ plugin of ImageJ/Fiji. To this aim, the fiber axis was set to 0°, and angles were measured. With the same image processing program, the area covered by CD31^+^ cells was also quantified by setting regions of interest (ROI) in different fibers (*n* = 3) and then calculating the % area of each ROI over the total area covered by cells. Similarly, the capillary size was analyzed by measuring this dimensions (*n* = 20) from three different images of independent samples at each time point.

### 4.9 Statistical Analysis

All measurements were performed in triplicate on at least three different samples produced from different cell cultures and tested independently. Data are reported as mean values ± standard deviation. A two-way ANOVA test and Tukey’s multiple comparison were run using GraphPad Prism v. 8.0 (GraphPad Software, USA), and differences are displayed as statistically significant when *p* ≤ 0.05. Statistically significant values are presented as *p ≤ 0.05, **p ≤ 0.01, ***p ≤ 0.001, and ****p ≤ 0.0001.

## Supporting Information Available

Additional experimental details including qualitative observations of the hydrogel precursors in terms of viscosity, quantitative compartmentalization of the hydrogel-based core–shell phases, and the effect of the fibrinogen core on the overall degradation of core–shell fibers; analysis of the core diameter by microcomputed tomography-based (μCT) volumetric reconstruction, focused ion beam-scanning electron microscopy (FIB-SEM) images of cell-laden fibers, influence of the MSC/HUVEC-laden fibers on the pH variability, and proof-of-concept of the microfluidic printing head (MPH) for the biofabrication of different vessel-like structures. Additional materials and methods details about the synthesis of Alginate-RGD and FITC-Alginate, FIB-SEM methodology, and pH variation analysis (PDF)
