# Supplementary material for: Engineering Biomimetic Microvascular Capillary Networks in Hydrogel Fibrous Scaffolds via Microfluidics-Assisted Co-Axial Wet-Spinning
Source: ACS Appl Mater Interfaces. 2024 Nov 20;16(48):65927–41. doi: 10.1021/acsami.4c15221 (PMC11622188; doi:10.1021/acsami.4c15221)
Supplement: Supplementary file 1 — am4c15221_si_001.pdf [file am4c15221_si_001.pdf]

Supporting Information

Engineering Biomimetic Microvascular Capillary Networks In Hydrogel Fibrous Scaffolds Via Microfluidics-Assisted Co-Axial Wet-Spinning

Alessia Paradiso[1], Marina Volpi[1], Diana C. Martinez[1], Jakub Jaroszewicz[1], Marco Costantini[2*], Wojciech Swieszkowski[1*]

[1] Faculty of Materials Sciences and Engineering, Warsaw University of Technology, 02-507 Warsaw, Poland

[2] Institute of Physical Chemistry, Polish Academy of Sciences, 01-224 Warsaw, Poland

***Corresponding Author**:

Prof. Wojciech Swieszkowski, wojciech.swieszkowski@pw.edu.pl

Dr. Marco Costantini, mcostantini@ichf.edu.pl

1. Results

1.1 Characterization of hydrogel-based materials

Alginate and fibrinogen were tested in their pre-hydrogel form to qualitatively evaluate their viscosity. Although they both possessed Newtonian-like rheological properties, the alginate pre-polymer formulation showed a higher viscosity than fibrinogen solution (45-fold higher). In **Figure S1**, a qualitative difference upon rotation of the vial (90° - 0°) can be appreciated, whereas such difference was detected by measuring the angle formed by the pre-polymer formulations once the vial reached the horizontal position (i.e., 0°) and recorded for a timeframe of 4 s.

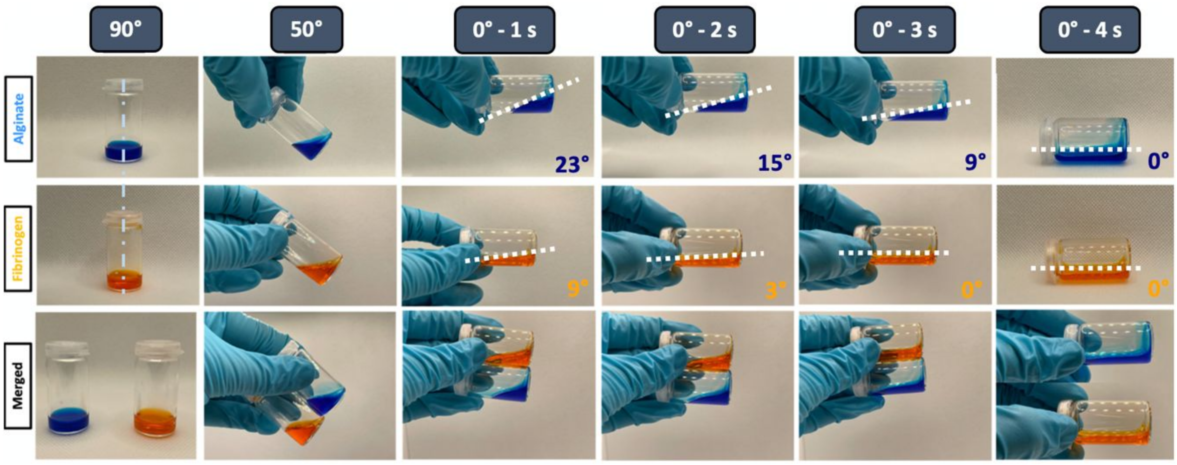

Figure S1. Qualitative assessment of the hydrogel precursors in terms of viscosity. 3% w/v alginate (shell - blue) and 1.4% w/v fibrinogen (core - orange) pre-polymer formulations were color-dyed (food-grade powder) and tested to inspect the different behavior of the liquid solutions at room temperature, by immediately rotating the respective glass vials up to 90° and considering a subsequent time span of 4 s. The dash line (white) spots the slope referred to the horizontal position.

1.2 Optimization of wet-spinning parameters and fiber compartmentalization characterization

1.2.1 Compartmentalization of the hydrogel-based core-shell phases

The coupling of different volumetric flow rates for the simultaneous wet-spinning of core and shell compartments allowed for tailoring the most suitable Q_s/Q_c combination for cell experiments. Although the total flow rate ($Q = Q_s + Q_c$) was set at 480 µl/min, the different couplings contributed to either reduced or increased core diameter. More in detail, the set $Q_s = 400/Q_c = 80$ µl/min produced fibers with a reduced core diameter, and in turn an increased wall thickness) than $Q_s = 320/Q_c = 160$ µl/min. The control of such features could be pivotal due to the potential influence of the wall thickness in regulating nutrients and oxygen exchange, as well as waste removal, throughout the shell compartment towards the core, thus potentially compromising cell viability or functionality.

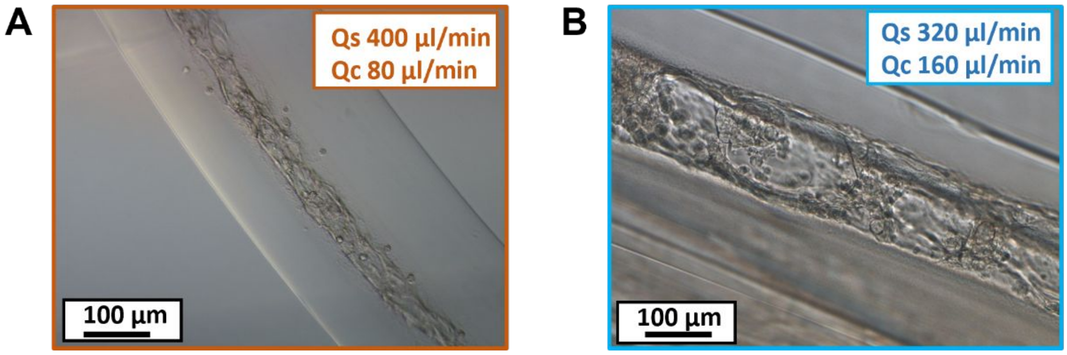

Figure S2. Flow rates optimization for the wet-spinning of cell-laden hydrogel fibers. Dimensional analysis of microfibers wet-spun at two different core-shell couplings of flow rates (total volumetric flow rate $Q = Q_s + Q_c$: 480 µl/min) in terms of fiber diameter, core diameter, and wall thickness. Fibers were wet-spun with the flow rate couplings **A**) Q_s:400/Q_c:80 µl/min and **B**) Q_s:320/Q_c:160 µl/min, as an increased wall thickness can influence the nutrient exchange and waste removal throughout the shell. Scale bar: 100 µm

1.2.2 Influence of the fibrinogen core on the overall degradation of the core-shell fibers

Figure S3A shows the core-shell fiber configurations chosen to mimic different types of fibers (**Table S1**), aiming at understanding the role of fibrinogen in the overall fiber degradation process. The SA3_CA3 was designed to simulate a standard fiber wet-spun with alginate 3% (w/v) only. The SA3_CA1 simulates a core-shell fiber with a soft alginate core. The SA3_CF fibers represented the optimized core-shell structure with a fibrinogen core. The influence of the fibrinogen-based core on the overall degradation process was analyzed comparing the degradation of core-shell fibers based on the same alginate-based shell. **Figure S3B** displays how all conditions tested rapidly degraded within the first day of immersion in cell culture medium. Although the constructs stabilized in a plateau-like stage up to day 14, the SA3_CF fibers presented a significant higher degradation than the alginate-based fibers at 7 days of culture, with an increased difference peak at 21 days.

Table S1. Core-shell fiber configurations to mimic different types of fibers

**Tested core-shell fiber configuration**		**Simulated fiber configuration**	**Legend**
**Shell (w/v)**	**Core (w/v)**		
Alginate 3%	Alginate 3%	Alginate-based homogenous fiber	SA3_CA3
Alginate 3%	Alginate 1%	Alginate-based core-shell fiber	SA3_CA1
Alginate 3%	Fibrinogen 1.4%	Multi-material core-shell fiber	SA3_CF

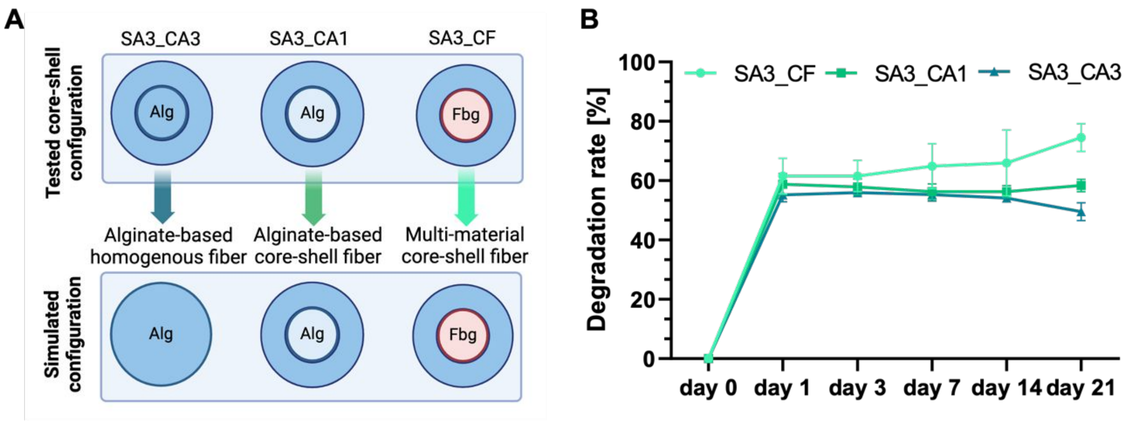

Figure S3. Degradation process of core-shell fibers characterized by an alginate-based (3% w/v) shell (SA3), while varying the core compartment. Legend: Alginate - Alg; Fibrinogen - Fbg. SA3_CF: core - fibrinogen 1.4% (w/v), SA3_CA1: core - alginate 1% (w/v), SA3_CA3: core - alginate 3% (w/v). A) Configuration of tested conditions and related simulation of the fiber (cross-section view). B) Degradation rate over a 3-week culture time.

1.3 Microstructural evaluation of cell-laden microfibers

The 3D µCT volumetric reconstruction allowed to analyze the core diameter of MSC/HUVEC-laden fibers after 7, 14, and 21 days of culture. Compared to the first week, the core of the fibers significantly increased after 14 days, as a result of both hydrogel swelling and cell proliferation. Afterwards, a reduced core was detected after 21 days of culture, confirming the role of fibrinogen degradation on the fiber architecture.

The top view of the specimens analyzed by FIB-SEM revealed a smooth surface of the fibers, as a result of the typical low porosity of alginate hydrogels. The cross sections of the hydrogel-based cell-laden fibers displayed the fibrin porosity along with cell densification areas as clearly visible at day 21. The dehydration process used for the SEM preparation led to a slight distortion of the hollow fibers. Similarly, low beam aperture size potentially contributed to the fiber damage, influencing the scanning process.

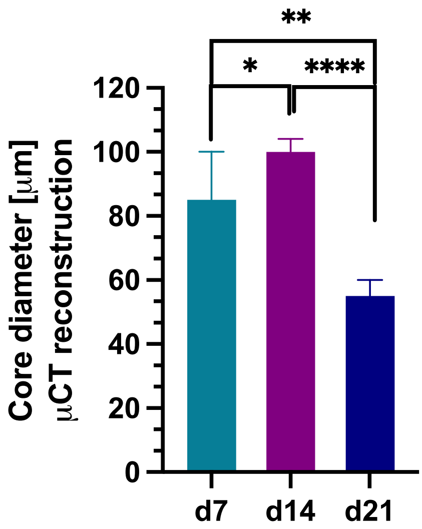

Figure S4. Analysis of the core diameter by µCT volumetric reconstruction. Core diameter of the 3D scanned hydrogel-based MSC/HUVEC-laden fibers at 1-, 2-, and 3-week culture time. Significant differences: *$p < 0.05$, **$p < 0.01$, ***$p < 0.001$, and ****$p < 0.0001$.

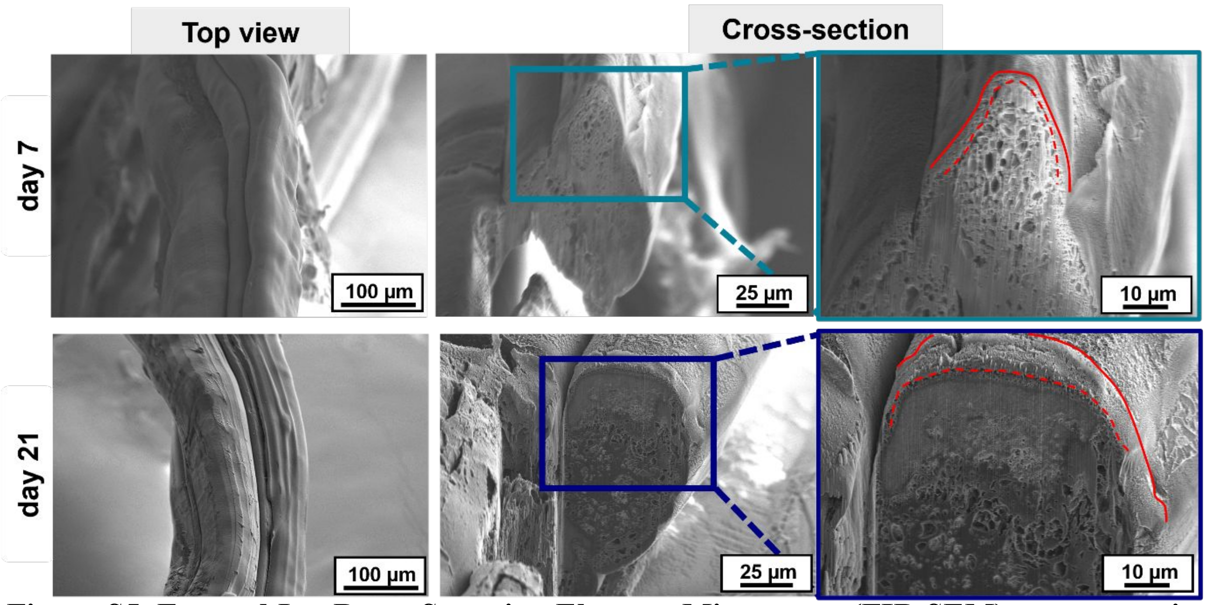

Figure S5. Focused Ion Beam Scanning Electron Microscopy (FIB-SEM) representative images of cell-laden fibers depicted after 7 and 21 days of culture. Fibers were imaged on top view and then FIB milled to detect the microstructure from the cross section of the constructs. Cross section insets were depicted with red lines to highlight the shell compartment: solid - outer wall, dash - inner wall. Scale bar: 100 µm (top view), 25 µm (cross section), and 10 µm (zoom-in).

1.4 Morphological analysis of the cell-laden bundles

To investigate the effect of the MSC/HUVEC-laden fibers on the cell culture medium conditions, the pH dynamics was evaluated over the 3-week time. The interaction between MSC and HUVEC within the 3D hydrogel scaffolds can create a dynamic and balanced environment that can mitigate extreme pH changes, promoting a more stable culture medium pH.

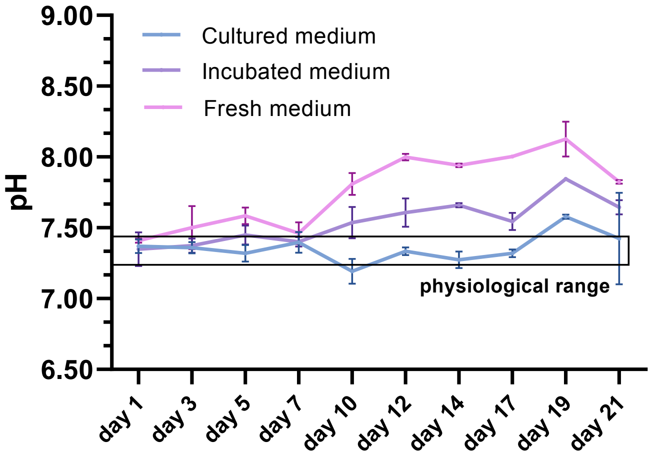

Figure S6. Influence of the MSC/HUVEC-laden fibers on the pH variability. The evaluation was conducted up to day 21 of culture. Legend: cultured medium - medium collected from cultured cell-laden fibers; incubated medium - medium cultured without 3D samples; fresh medium - medium from the stock stored at 4° C. The blue rectangle refers to physiological

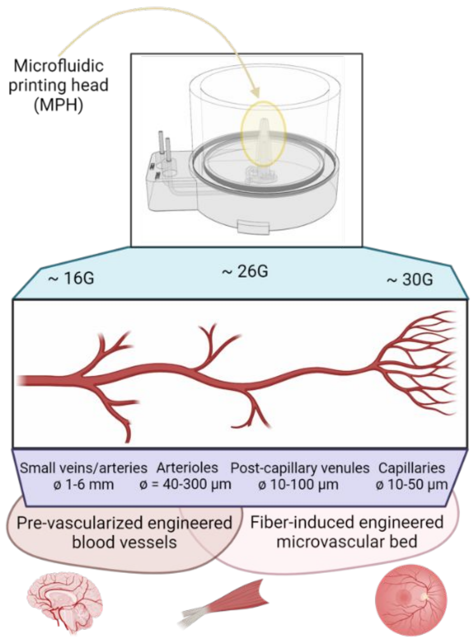

Figure S7. Potential use of the microfluidic printing head (MPH) for the biofabrication of different vessel-like structures. Native diameters of anatomically different types of blood vessels can be achieved by coupling the 3D rotary wet-spinning system with a customizable MPH in terms of core-shell configuration size.

pH.

2. Materials and Methods

2.1 Synthesis of Alginate-RGD

Arginine–glycine–aspartic acid (RGD) tripeptide was introduced into sodium alginate via chemical modification, following previously published protocols. Sodium alginate (1% w/v) was stirred in 2-ethanesulfonic acid (MES) buffer (0.1 M MES, 0.3 M NaCl, pH 6.5) until fully dissolved. Then, also EDC and NHS were added within the alginate-MES buffer to activate the carboxyl groups of alginate, stirring for 30 min at RT to allow the reaction to proceed. Upon activation, lyophilized GRGDSP peptides (ProteoGenix, France) were added to the solution (20 mg/g alginate) and let reacting overnight at room temperature allowing for the conjugation reaction to occur. Subsequently, the solution was purified from unreacted EDC, NHS and RGD by performing dialysis against deionized water (3.5 kDa MWCO - Merck, Germany) at 4 °C, changing water once per day for 3 days. Finally, it was lyophilized and stored at -20 °C until further use.

2.2 Synthesis of FITC-Alginate

FITC-Alginate was synthesized via a dual-step reaction. First, an aqueous solution of 1% (w/v) sodium alginate was mixed with 1-Ethyl-3-(3-dimethylaminopropyl)carbodiimide (EDC, 50 mg) and N-hydroxysuccinimide (NHS, 30 mg) to activate the carbonyl groups of sodium alginate in sodium acetic buffer (pH 4.9) for 30 min under stirring condition at room temperature (RT). Then, 1,6-diaminohexane (60 mg) was added to the solution and let stir for other 4 hours of reaction at RT. Afterward, the solution was precipitated in isopropyl alcohol (2-propanol), which was previously kept at 4 °C, to remove the unreacted diamine (RT, stirring condition). Then, the alginate derivative was centrifuged to obtain a pellet cleared from 2-propanol. Subsequently, the alginate-amine derivative was poured into a sodium carbonate buffer ($NaHCO_3$, pH 9.0) to react with fluorescein isothiocyanate (FITC, 1 mg) for 4 hours, protected from light (RT, stirring conditions). Afterward, the mixture was precipitated in acetone, then centrifuged and deionized water was added to product until fully dissolved (RT, stirring conditions). Finally, the solution was dialyzed against deionized water (3.5 kD MWCO - Merck, Germany) for 3 days at 4 °C and then lyophilized for up to 4 days.

2.3 Synthesis of FITC-Alginate

Viscosity: the biomaterial inks were prepared and poured into two different glass vials. A food-grade die was added to clearly distinguish the solutions while performing the qualitative test. Vials were rotated to reach a 90° position (i.e., horizontal position), to allow the ink for covering the vial wall. Image frames were taken at different time points up to 4 s.

2.4 Focused Ion Beam Scanning electron microscopy (FIB-SEM)

Prior to imaging, scaffolds at selected time points (i.e., 7, 21 days) were dehydrated using 50%, 70, 90%and 100% concentrated ethanol solutions (POCH, Poland) for 15 min each. Then, the

constructs were immersed in hexamethyldisilazane (HMDS, Merck, Germany) for other 15 min and then let dry in a fume hood overnight. Subsequently, samples were sputtered with a 10 nm layer of gold at 10kV (EM SCD 500, Leica Systems, Germany) as a protective layer to minimize the damage during the FIB milling steps. At an acceleration voltage of 5kV, top view SEM images were taken at three different magnification fields to inspect the microstructure. Additionally, cross-section micrographs of the fibers were analyzed using a dual beam system combining a FIB with the SEM (Hitachi, NB-5000 dual beam, Japan). The fiber cross sections were FIB-milled at 40kV acceleration voltage and low probe current (0.58 - 0.73 nA).

2.5 pH variation analysis

The pH of the cell culture medium (CCM) was measured using a pH meter (SevenMulti, Mettler Toledo, Ohio, USA) with an InLab® Flex-Micro probe after each CCM change for up to 21 days. Tested conditions were cultured medium (i.e., medium collected from 3D cultured cell-laden hydrogel bundles), incubated medium (i.e, incubated medium cultured without 3D samples), and fresh medium (i.e., medium from the stock stored at 4° C).
